# Multi-Layer Transcriptome Analysis Uncovers Molecular Signatures of Coliform Mastitis in Lactating Cows

**DOI:** 10.3390/ijms27167134

**Published:** 2026-08-09

**Authors:** Yamini Sri Sekar, Kuralayanapalya Puttahonnappa Suresh, Shivasharanappa Nayakvadi, Bramhadev Pattnaik, Azhahianambi Palavesam, Nagendra Nath Barman, Hariprasad Thippeswamy, Varsha Ramesh, Sharanagouda Shiddanagouda Patil

**Affiliations:** 1 Indian Council of Agriculture Research (ICAR)-National Institute of Veterinary Epidemiology & Disease Informatics (NIVEDI), Bengaluru 560119, Karnataka, India; yaminisekar0616@gmail.com (Y.S.S.);; 2 Institute of Veterinary Science and Animal Husbandry (IVSAH), Siksha O Anusandhan (SOA) University, Bhubaneswar 751029, OdishaIndia, India; 3 Translational Research Platform for Veterinary Biologicals Centre for Animal Health Studies, Tamil Nadu Veterinary and Animal Sciences University, Chennai 600051, Tamil Nadu, India; 4 Department of Veterinary Microbiology, College of Veterinary Science, Assam Agricultural University, Guwahati 785013, Assam, India

**Keywords:** *E. coli* mastitis, WGCNA, gene regulation, miRNA, machine learning

## Abstract

Bovine mastitis caused by *Escherichia coli* remains one of the most economically significant diseases in the dairy industry. This study performed a multi-layer transcriptome analysis of publicly available microarray data (GSE15025) derived from 15 German Holstein-Friesian heifers experimentally inoculated with *E. coli*, yielding 30 microarray samples across three GEO datasets (GSE15019, GSE15020, GSE15022) representing infected quarters at 6 h and 24 h post-inoculation and neighboring uninfected quarters at 24 h. Using an analytical framework combining WGCNA, differential gene expression profiling, PPI network construction, SVM classification, and computational miRNA target prediction, three significant co-expression modules were identified blue (367 genes, 6 h), turquoise (3602 genes, 24 h), and yellow (193 genes, 24 h neighboring tissue) alongside 21, 1570, and 250 differentially expressed genes respectively. Cross-referencing WGCNA and PPI analyses identified STAT3, JUN, and RAC2 as high-confidence hub gene candidates with the strongest convergent computational support. An SVM classifier achieved 87.5% accuracy (sensitivity = 100%, specificity = 75%) in distinguishing infected from control samples. Computational miRNA predictions suggested putative regulatory interactions requiring experimental confirmation. As a purely computational study, experimental validation remains a necessary future step; however, these findings provide a systematic characterization of candidate molecular mechanisms and potential biomarker targets for *E. coli*-induced bovine mastitis.

## 1. Introduction

Bovine mastitis remains one of the most prevalent and economically significant diseases in the global dairy industry, severely affecting milk yield, quality, and animal welfare [1,2]. It is characterized by inflammation of the mammary gland primarily resulting from microbial infection and is broadly classified in veterinary practice as clinical mastitis characterized by visible signs of inflammation, systemic illness, and abnormal milk or subclinical mastitis, which lacks overt clinical signs but causes significant production losses and is detected through elevated somatic cell count [3,4]. The disease imposes a considerable economic burden on the dairy sector, with annual losses estimated at approximately 2 billion USD in the United States and 526 million USD in India, of which subclinical mastitis accounts for nearly 70% of the total [2].

A wide range of microorganisms have been implicated in bovine mastitis. Contagious pathogens such as *Staphylococcus aureus*, *Streptococcus agalactiae*, and *Streptococcus dysgalactiae* are primarily transmitted during milking or by direct animal contact [5,6]. Environmental pathogens including *Escherichia coli*, *Klebsiella pneumoniae*, and *Streptococcus uberis* are frequently associated with infections originating from contaminated bedding, water, or soil [7,8]. Among these, *E. coli* is recognized as a major causative agent of acute bovine mastitis, accounting for approximately 16–35% of clinical mastitis cases depending on the region and study [9], and is characterized by rapid onset and severe inflammatory manifestations. Mammary pathogenic *E. coli* (MPEC) strains possess virulence determinants that facilitate bacterial colonization and immune evasion in mammary tissue [10,11], triggering extensive epithelial damage and gland dysfunction through activation of innate immune signaling pathways.

Recent advances in transcriptomics have helped clarify the molecular mechanisms underlying the host response to bovine mastitis. Ghahramani et al. [12] analyzed multiple microarray datasets and identified 715 differentially expressed genes (DEGs), with hub genes including PRDX5, MAPK6, CD53, and ACTN4 linked to host immune defense and stress regulation. Younis et al. [13] integrated datasets GSE15019 and GSE15020 to reveal conserved immune response signatures, while Naserkheil et al. [14] expanded the analysis to include GSE15022, uncovering key regulatory genes including CXCR1, HCK, IL1RN, MMP9, S100A9, and SOCS3. Using RNA-seq data, Bakhtiarizadeh et al. [15] applied Weighted Gene Co-expression Network Analysis (WGCNA) and identified immune-related modules and central regulators including STAT1, NFKBIA, HCK, and TLR6. Studies on *S. aureus* and *E. coli* infections have further highlighted genes such as IL1B, TNF, CXCL8, and HCK as critical mediators of cytokine and chemokine signaling [16]. Computational analyses have additionally suggested putative post-transcriptional regulation through predicted miRNA–mRNA interaction networks [17], though experimental validation of these interactions remains limited. Together, these studies point to a shared molecular framework for mastitis involving the NF-κB, MAPK, PI3K–AKT, and IL-17 signaling pathways.

Despite these advances, two important research gaps remain. First, relatively few studies have simultaneously integrated all three major publicly available *E. coli* mastitis microarray datasets GSE15019 (6 h post-inoculation), GSE15020 (24 h post-inoculation), and GSE15022 (24 h neighboring uninfected tissue) into a unified analysis, limiting the identification of gene expression signatures consistently represented across experimental conditions. Second, relatively few studies have combined differential gene expression analysis with co-expression network modeling in a cross-validated framework, which constrains the identification of hub genes that are both statistically significant and biologically central to mastitis pathophysiology.

To address these gaps, the present study integrates all three datasets and applies a multi-method transcriptomic analysis framework combining differential expression analysis, WGCNA, protein–protein interaction (PPI) network construction, support vector machine (SVM)-based classification, and computational miRNA target prediction using the miRNet platform. The miRNA analysis is based entirely on database-derived target prediction and does not incorporate experimental miRNA expression data; these interactions should therefore be interpreted as putative regulatory associations requiring experimental confirmation. This framework aims to provide a systematic transcriptomic characterization of the host response to *E. coli*-induced bovine mastitis and identify candidate hub genes and regulatory targets that may inform future experimental investigations and resistance breeding strategies in dairy cattle.

## 2. Results

### 2.1. Construction of Weighted Gene Co-Expression Network and Identification of Key Modules

**Figure 1:**
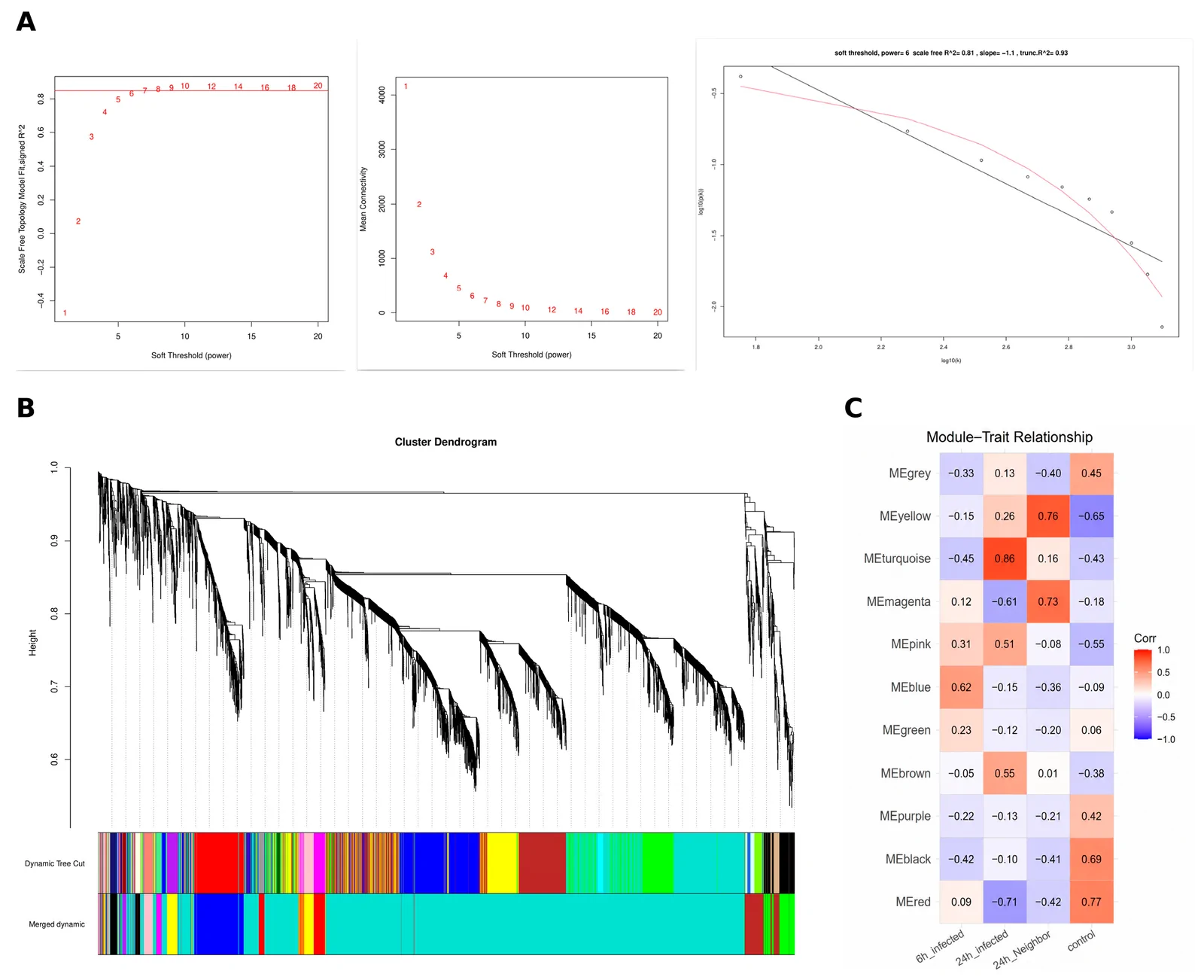
Weighted Gene Co-expression Network Analysis (WGCNA) for module detection and trait association. (**A**) Selection of soft-thresholding power for network construction. Scale-free topology fit index (R^2^) as a function of soft-threshold power. Middle: Mean connectivity plot indicating network sparsity across powers. Scale-free topology model fit demonstrating optimal power selection to establish biologically meaningful co-expression relationships. (**B**) Gene clustering dendrogram based on topological overlap matrix (TOM) dissimilarity. Dynamic tree cutting identified initial gene modules (upper colored bars), which were subsequently merged based on eigengene similarity to form final consensus modules (lower colored bars). (**C**) Module–trait relationship heatmap showing Pearson correlation coefficients between module eigengenes and experimental groups (control, 6 h infected, 24 h infected and 24 h neighbor). Red shading denotes positive correlation and blue indicates negative correlation, with intensity proportional to correlation strength. Significant module–trait associations highlight key gene sets potentially involved in the host response across infection stages.

To explore gene co-expression patterns associated with bovine mastitis, a weighted gene co-expression network was constructed using the expression profiles of 5000 genes derived from 30 samples in the GSE15025 dataset. The soft-thresholding power (β) was optimized to satisfy the scale-free topology criterion, ensuring that the resulting network reflected real biological organization. A value of β = 6 was chosen, which resulted in a scale-free topology fit index (R^2^) of 0.81 (Figure 1B), indicating that the network structure closely followed the expected scale-free property commonly observed in biological systems. Hierarchical clustering of genes based on expression similarity produced a well-defined dendrogram (Figure 1A), where genes with similar expression profiles were grouped together. This analysis resulted in the identification of eleven distinct co-expression modules, each represented by a unique color (Figure 1C). Modules exhibiting high eigengene similarity were subsequently merged using a module dissimilarity threshold (MEDissThres) of 0.25, yielding a refined and biologically coherent set of modules. Genes that did not cluster into any specific module were grouped under the gray module, indicating weak or inconsistent expression relationships with other genes. These unassigned genes were excluded from downstream analyses to maintain focus on robust and biologically meaningful modules. Overall, the resulting network structure provided a solid foundation for linking specific gene modules to mastitis-associated phenotypes in subsequent analyses.

### 2.2. Module–Trait Correlation Analysis

The relationship between gene co-expression modules and *E. coli* infection status was examined to identify modules significantly associated with different stages of infection. Based on the correlation analysis, the blue module showed a strong positive correlation with the 6 h post-infection stage (r = 0.62, *p* = 2.4 × 10^−4^), indicating that genes in this module are actively involved in the early immune response. The turquoise module exhibited the highest positive correlation with the 24 h post-infection stage (r = 0.86, *p* = 1.07 × 10^−9^), suggesting that it represents genes participating in sustained or late immune responses. Additionally, the yellow module was significantly correlated with the 24 h neighboring (uninfected) tissue condition (r = 0.76, *p* = 1.16 × 10^−6^), implying that genes in this module may be involved in local immune signaling or protective mechanisms adjacent to infected tissue. Based on these findings, the blue, turquoise, and yellow modules were identified as the key infection-associated modules and were selected for subsequent downstream analyses to explore their biological significance and hub gene composition.

### 2.3. Identification of Hub Genes Within Significant Modules

**Figure 2:**
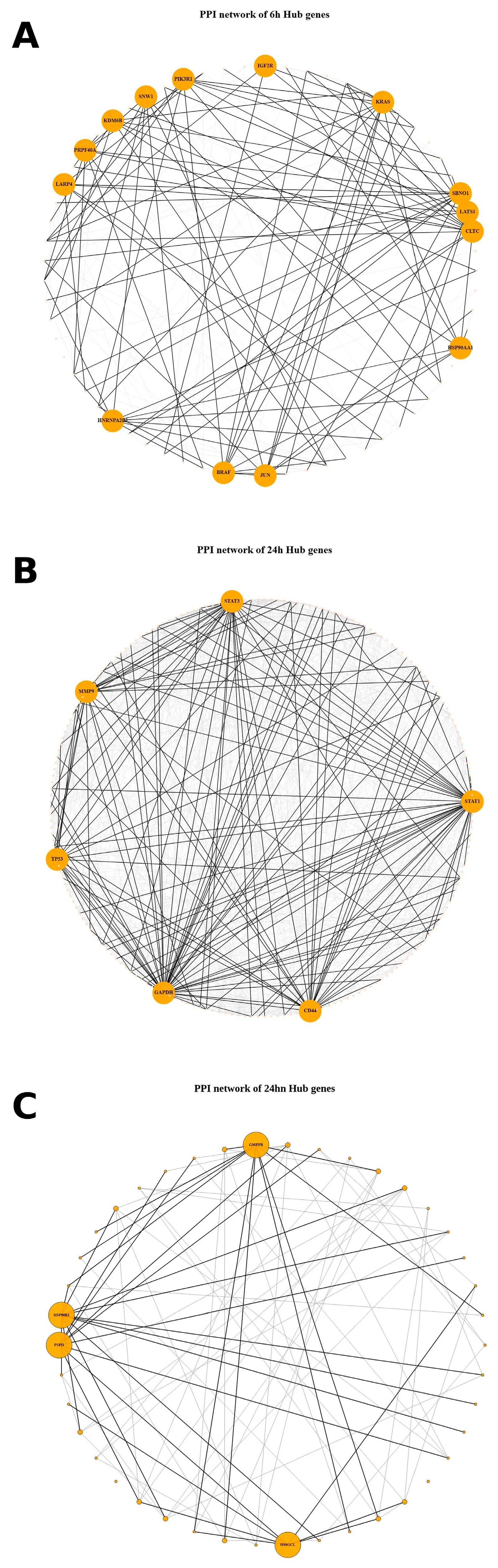
Protein–protein interaction (PPI) networks of hub genes identified across infection stages. PPI networks illustrating the connectivity patterns of (**A**) 6 h hub genes, (**B**) 24 h hub genes, and (**C**) 24 h neighbor hub genes derived from integrative differential expression and co-expression analyses. Nodes represent hub genes, with node size proportional to their degree of connectivity, while edge thickness reflects interaction strength. The progressive changes in network density and centrality highlight dynamic remodeling of host molecular interactions during *E. coli*-induced mastitis and suggest stage-specific regulatory nodes critical for immune activation and stress adaptation.

As summarized in Table S1, the blue, turquoise, and yellow modules contained 367, 3602, and 193 genes respectively. To identify the most significant genes within each module, thresholds of Module Membership (MM) > 0.80 and Gene Significance (GS) > 0.50 were applied, yielding candidate gene sets of 68, 1132, and 67 genes for the blue, turquoise, and yellow modules respectively (Table 1). These candidate genes were imported into the STRING database to construct protein–protein interaction (PPI) networks, visualized using Cytoscape v3.10.3 (Figure 2). Using the CytoHubba plug-in, top connected nodes were ranked by degree, identifying 14, 6, and 4 PPI-derived hub genes in the blue, turquoise, and yellow modules respectively (Table S2).

Cross-referencing WGCNA candidate genes with PPI-derived hub genes revealed overlapping genes in all three modules 5 in the blue module, 6 in the turquoise module, and 1 in the yellow module (Table 2). To confirm that these overlaps were greater than expected by chance, a hypergeometric test was performed. Significant enrichment was observed for the blue module (*p* = 7.30 × 10^−7^) and turquoise module (*p* = 1.33 × 10^−4^), confirming robust concordance between co-expression and PPI network analyses. The yellow module overlap did not reach statistical significance (*p* = 0.0525), likely reflecting the smaller gene set size. Genes consistently identified across both analytical frameworks are designated high-confidence hub genes, as their convergent detection through two independent and mechanistically distinct approaches—co-expression topology and protein interaction network centrality—reduces the likelihood of false-positive identification. Significantly correlated modules were further subjected to GO and KEGG enrichment analyses to characterize the biological functions of the identified hub genes.

### 2.4. Expression Profiling and Identification of Differentially Expressed Genes (DEGs) Differential Expression Analysis Was Performed to Identify Genes Significantly Regulated During E. coli Infection at Different Time Points

At the 6 h infection stage, a total of 21 differentially expressed genes (DEGs) were identified, including 15 upregulated and 6 downregulated genes (*p* < 0.1, |FC| > 2.0). In the 24 h infected quarter, a much stronger transcriptional response was observed, with 1570 DEGs, of which 1124 were upregulated and 446 were downregulated under the same statistical thresholds. In contrast, the 24 h neighboring (uninfected) quarter showed 250 DEGs, comprising 152 upregulated and 98 downregulated genes (*p* < 0.05, |FC| > 2.0). Subsequently, the hub genes identified from these DEGs were analyzed using the STRING database to construct PPI networks (Table 3). The top hub genes obtained from co-expression network analysis (WGCNA) and those from differential expression analysis revealed several hub genes, which are listed in Table 4. These genes represent key molecular regulators consistently identified by both analytical approaches and are likely to play central roles in the immune and inflammatory response to *E. coli*-induced bovine mastitis.

### 2.5. Temporal Functional Enrichment Analysis During E. coli Infection

**Figure 3:**
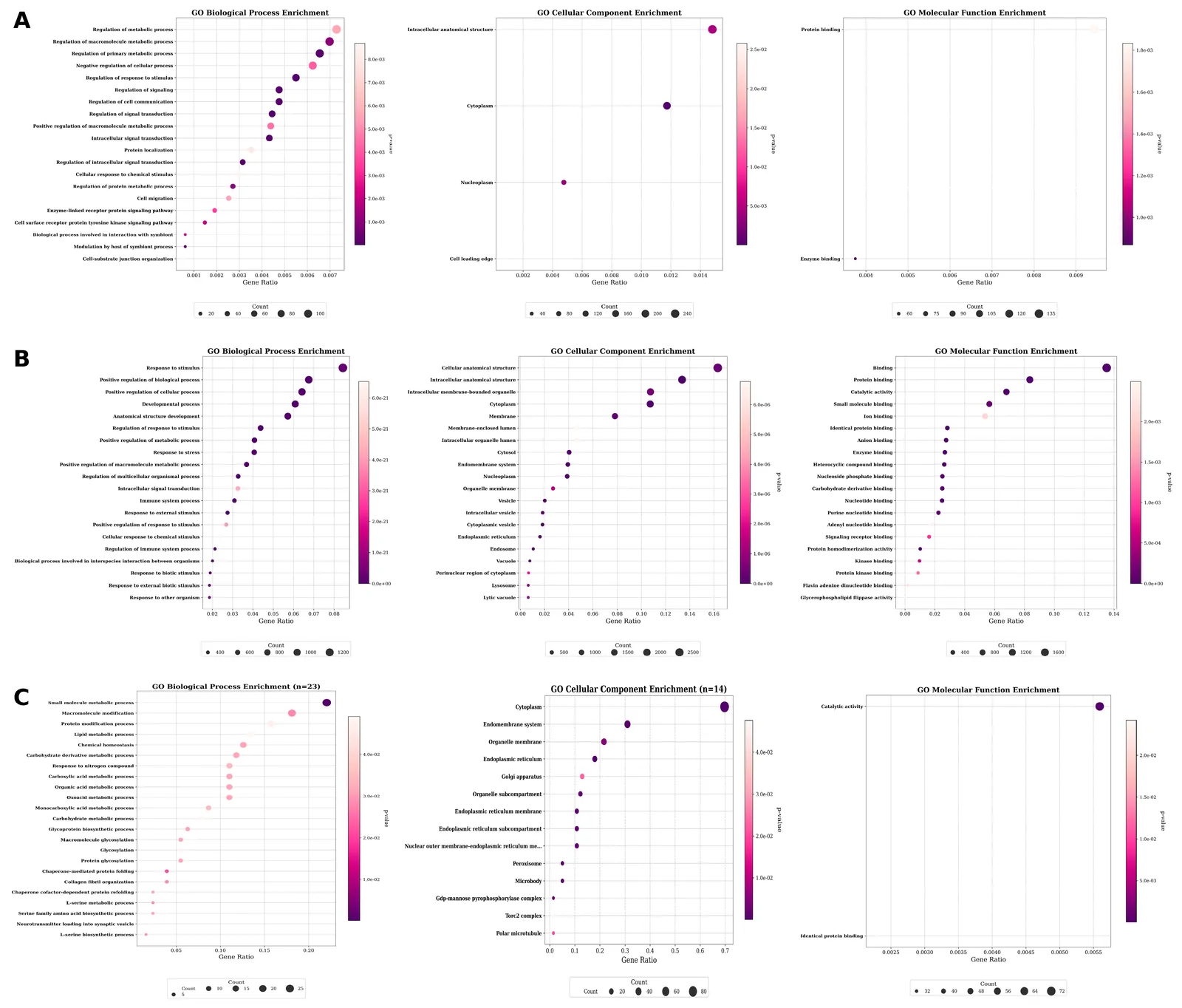
GO enrichment analysis of differentially expressed genes at (**A**) 6 h, (**B**) 24 h, and (**C**) 24 hn.

Gene enrichment analysis revealed distinct time-dependent and tissue-specific patterns of host response across the 6 h (6 h), 24 h (24 h), and 24 h neighboring (24 hn) samples (Figure 3). At 6 h post-infection, enriched biological processes were mainly linked to metabolic regulation, response to stimulus, and intracellular signaling, indicating rapid activation of stress and pathogen-responsive pathways. These processes were largely localized to the cytoplasm and intracellular compartments, reflecting early transcriptional and signaling activity. By 24 h, enrichment shifted toward strong immune and stress responses, including positive regulation of cellular and biological processes, developmental pathways, and anatomical structure organization. Enriched cellular components such as the endomembrane system, endoplasmic reticulum, vesicles, and lysosomes highlight increased protein trafficking, secretion, and degradation during immune activation. In the 24 h neighboring tissue, enrichment focused on metabolic homeostasis, particularly small molecule metabolism, lipid metabolism, and macromolecule modification, suggesting that adjacent tissues maintain energy balance and stress tolerance rather than immune activation. Module-specific analyses supported these trends. The blue module (6 h) was enriched in ErbB (FDR = 2.4 × 10^−2^) and mTOR (FDR = 2.4 × 10^−2^) signaling, as well as focal adhesion and cancer-related pathways, reflecting early cell survival and stress adaptation mechanisms. The turquoise module (24 h) showed strong enrichment for immune-related pathways, including Malaria, Pertussis, Leishmaniasis, HIF-1 signaling, and leukocyte migration (FDR < 1.9 × 10^−5^), indicating robust inflammatory and hypoxia responses. Conversely, the yellow module (24 hn) was enriched in ferroptosis, cysteine and methionine metabolism, and PPAR signaling, consistent with metabolic regulation and redox balance in uninfected neighboring tissues. Overall, these results highlight a temporal shift from early signaling to strong immune activation at 24 h, while neighboring tissues maintain metabolic stability to counter infection-induced stress (Table 5).

The turquoise module (24 h) showed significant enrichment for several KEGG pathways that, while named after infectious diseases (Malaria: FDR = 1.7 × 10^−6^; Pertussis: FDR = 3.6 × 10^−6^; Leishmaniasis: FDR = 8.1 × 10^−6^), share conserved innate immune signaling components relevant to bacterial infection. These pathways converge on core inflammatory mediators including Toll-like receptor (TLR) signaling, NF-κB activation, pro-inflammatory cytokines (TNF, IL-6, IL-1β), and complement cascade components—all of which are well-established effectors of the bovine mammary immune response to *E. coli*. Their enrichment in the turquoise module therefore reflects the activation of shared innate immune machinery rather than any direct biological association with these diseases. This interpretation is further supported by the concurrent enrichment of mechanistically specific pathways: HIF-1 signaling (FDR = 1.7 × 10^−5^), which mediates hypoxia-driven inflammatory gene expression, and leukocyte transendothelial migration (FDR = 1.9 × 10^−5^), reflecting the recruitment of immune cells to the site of infection—both of which are canonical features of acute bacterial mastitis.

Bubble plots represent Gene Ontology (GO) enrichment across three categories Biological Process (BP), Cellular Component (CC), and Molecular Function (MF) for (A) 6 h post-infection (6 h), (B) 24 h post-infection (24 h), and (C) 24 h neigbour gene set (24 hn). Bubble size indicates the number of enriched genes, and color intensity reflects adjusted *p*-values. At 6 h, enriched pathways primarily involved early immune activation and metabolic regulation. By 24 h, enrichment expanded to include processes associated with inflammatory signaling, cytokine responses, mitochondrial activity, and protein-binding functions. The 24 hn genes highlighted distinct pathway signatures representing late-stage cellular reorganization and immune–stress adaptation. Together, these GO profiles depict the temporal progression of host molecular responses during *E. coli*-induced mastitis.

### 2.6. Predictive Validation of Hub Genes Through SVM Classification

To further evaluate the biological relevance and predictive potential of the identified hub genes (RAC2, PIK3CA, PIK3CD, EGFR, WDR46, CDK1, UTP18, NAT10, MAPK14, MAPK13, GNAI3, GNAI2, EIF2S1, EBNA1BP2, WDR75, BRAF, SNW1, LATS1, LARP4, JUN, GAPDH, STAT1, STAT3, CD44, TP53, MMP9, and HMGCL), a machine learning (ML)-based classification analysis was performed. The goal was to assess how effectively these genes could distinguish between infected and control samples. Among the tested classifiers, the Support Vector Machine (SVM) model demonstrated the best performance, achieving an accuracy of 87.5%, with 100% sensitivity and 75% specificity (Table 6). The balanced accuracy of 87.5% further confirmed the robustness of the model in correctly classifying both infected and control samples. These findings indicate that the selected hub genes possess strong discriminatory power and play a significant role in distinguishing infection status. Their consistent contribution to accurate classification provides additional support for their biological importance in *E. coli*-induced bovine mastitis and highlights their potential as reliable biomarkers for disease detection and prediction.

### 2.7. miRNA-Mediated Regulation of Mastitis-Associated Hub Genes

To explore putative post-transcriptional regulatory mechanisms influencing mastitis-associated gene expression, the identified hub genes were subjected to miRNA target prediction analysis using the miRNet platform. It is important to note that all miRNA–mRNA associations reported here are computationally predicted based on sequence complementarity and database-curated target algorithms, and have not been experimentally validated; these findings should therefore be interpreted as candidate regulatory interactions warranting further investigation. Among the hub genes, MAPK14 was predicted to be targeted by the largest number of miRNAs (*n* = 20), including bta-miR-339a, bta-miR-370, bta-miR-381, bta-miR-489, bta-miR-149-5p, and bta-miR-7865, suggesting its potential role as a regulatory target in stress-activated signaling and cytokine-mediated inflammation. Similarly, STAT3 was computationally associated with 18 miRNAs, including bta-miR-21-5p, bta-miR-17-5p, bta-miR-320a, bta-miR-342, and bta-miR-485, consistent with its known importance in immune signaling though the functional relevance of these predicted interactions in the bovine mammary context remains to be confirmed. GNAI2 showed predicted associations with 16 miRNAs including bta-miR-138, bta-miR-615, and bta-miR-2881, pointing to its possible involvement in receptor-mediated signaling networks. JUN and RAC2 were predicted to be targeted by 12 and 8 miRNAs, respectively, suggesting potential post-transcriptional regulation of transcriptional and cytoskeletal remodeling processes during infection. Notably, certain miRNAs including bta-miR-1777a/b, bta-miR-2433, and bta-miR-664a were predicted to target multiple hub genes simultaneously, suggesting they may act as candidate hub miRNAs with broad regulatory coverage across mastitis-associated pathways. However, this designation is based solely on target prediction overlap and should not be interpreted as evidence of coordinated regulatory activity without experimental confirmation. Validation through luciferase reporter assays, miRNA overexpression or inhibition studies, or Ago2-CLIP sequencing would be necessary to establish direct regulatory relationships between these miRNAs and their predicted target hub genes. The complete list of predicted miRNA–mRNA interactions is presented in Table 7.

## 3. Discussion

### 3.1. Multi-Method Transcriptomic Framework for Hub Gene Identification

Mastitis in bovine caused by *Escherichia coli* is an acute inflammatory infection of the udder tissue, frequently emerging during the dry period or early lactation, and remains one of the most economically significant diseases in dairy cattle worldwide due to its impact on milk yield, milk quality, and animal welfare [18]. Despite its prevalence, the molecular mechanisms governing the host transcriptional response to *E. coli* infection remain incompletely understood, particularly with respect to the temporal dynamics of immune activation across different stages of infection. To address this, the present study employed a multi-method transcriptomic analysis framework, systematically combining differential expression analysis, weighted gene co-expression network modeling, protein–protein interaction network construction, machine learning-based classification, and computational miRNA target prediction. By cross-referencing results across these complementary analytical methods, highly interconnected hub gene candidates were identified through convergent evidence across multiple independent analytical frameworks, reducing reliance on any single method and strengthening the reliability of the identified biomarker candidates.

### 3.2. Early Infection Response—6 h Hub Genes (Blue Module)

The blue module showed significant positive correlation with the 6 h post-infection stage (r = 0.62, *p* = 2.4 × 10^−4^), containing 367 genes of which 68 met the MM and GS candidate thresholds. SNW1 (MM = 0.941, GS = 0.619), LATS1 (MM = 0.907, GS = 0.521), and LARP4 (MM = 0.961, GS = 0.556) were identified as WGCNA-derived hub gene candidates (Table 1), while JUN (degree = 48, betweenness = 3466) emerged as the top DEG-derived PPI hub gene at this time point (Table 4).

SNW1’s early-phase network position is consistent with its role in facilitating transcriptional elongation and splicing of inflammatory genes downstream of NF-κB signaling a pathway central to macrophage activation during the initial host response to bacterial infection [19,20]. Notably, SNW1 knockdown markedly reduces expression of key inflammatory mediators including IL-1, CCL2, and COX2 [19], suggesting its early identification reflects a role in priming the NF-κB-driven transcriptional cascade preceding full immune activation at 24 h. LATS1’s early-phase identification may reflect infection-induced activation of tissue integrity and cell survival mechanisms in mammary epithelial cells prior to full inflammatory escalation [21], consistent with the blue module’s enrichment in mTOR and ErbB signaling pathways (Table 5). LARP4, carrying the highest MM score among blue module candidates, supports the translational machinery required for the rapid immune gene expression program initiated within 6 h of *E. coli* infection through regulation of poly(A) tail length and interaction with PABPC1 [22]. JUN, as a component of the AP-1 transcription complex cooperating with NF-κB and STAT1, is consistent with its role in coordinating early host immune responses supported by Bi et al. [23], who confirmed significant c-Jun upregulation via RT-qPCR in mastitis-infected mammary tissues.

### 3.3. Peak Infection Response—24 h Hub Genes (Turquoise Module)

The turquoise module demonstrated the strongest positive correlation with the 24 h post-infection stage (r = 0.86, *p* = 1.07 × 10^−9^), representing the largest module with 3602 genes, of which 1132 met candidate thresholds. STAT1 (MM = 0.947, GS = 0.822), STAT3 (MM = 0.949, GS = 0.744), CD44 (MM = 0.937, GS = 0.898), TP53 (MM = 0.847, GS = 0.722), and MMP9 (MM = 0.801, GS = 0.979) were identified as WGCNA hub gene candidates (Table 1), while PIK3CD, MAPK14, MAPK13, GNAI2, GNAI3, EIF2S1, NAT10, CDK1, WDR46, EBNA1BP2, and WDR75 emerged as top DEG-derived PPI hub genes (Table 3).

STAT1 and STAT3, both central JAK-STAT pathway transcription factors, reflect sustained interferon- and cytokine-driven signaling during peak inflammation. STAT1 may modulate inflammation severity through induction of SOCS1 and SOCS3 [15,24], while STAT3 additionally predicted to be targeted by 18 miRNAs (Table 7)—governs acute-phase immune responses and lysosomal-mediated programmed cell death during mammary gland remodeling. Experimental support for STAT3 upregulation has been confirmed via RT-qPCR in bovine mammary epithelial cells [25] and during mammary involution [26]. CD44, carrying one of the highest GS scores in the module (GS = 0.898), reflects active neutrophil recruitment and adhesion dynamics at the infection site, with the CD44v10 variant specifically expressed on bovine milk neutrophils during mastitis [27,28]. MMP9, with the highest GS score among all WGCNA candidates (GS = 0.979), shows the strongest association with the 24 h infection phenotype, consistent with its markedly elevated expression during *E. coli* and *S. aureus* mastitis and its correlation with somatic cell count [29,30], further supporting its potential as a transcriptomic biomarker for mastitis severity. TP53 upregulation in the 24 h network likely represents a departure from normal lactation-phase suppression [31], reflecting infection-driven apoptotic tissue remodeling.

Among DEG-derived hub genes, PIK3CD (degree = 71) coordinates PI3K/AKT-mediated immune cell activation [21,32]; MAPK14 (degree = 59) and MAPK13 (degree = 58) reflect coordinated stress-kinase signaling consistent with the turquoise module’s HIF-1 and leukocyte migration enrichment (Table 5) [33,34,35,36]; GNAI2 and GNAI3 coordinate GPCR-mediated immune cell trafficking directly mirroring leukocyte trans-endothelial migration enrichment (FDR = 1.9 × 10^−5^) [12,37]; and EIF2S1, carrying the highest betweenness centrality among all DEG-derived hub genes (betweenness = 458,957), suggests a central coordinator role linking ER stress signaling to immune adaptation [37]. NAT10 and LARP4 support infection-induced translational stress responses [22,38], CDK1 may reflect infection-induced disruption of epithelial cell cycle progression [39], while WDR46, EBNA1BP2, and WDR75 likely reflect broader ribosomal biosynthesis upregulation to sustain immune protein synthesis demands, though direct mastitis-specific evidence remains lacking [40,41,42].

Although GAPDH exhibited the highest GS score among turquoise module candidates (GS = 0.950; Table 1), it was excluded from biological interpretation as its high network metrics likely reflect constitutive housekeeping expression rather than infection-specific regulation [43]. This highlights the importance of pre-filtering housekeeping genes prior to co-expression network construction in future analyses.

### 3.4. Neighboring Tissue Response—24 h Hub Genes (Yellow Module)

The yellow module was significantly correlated with the 24 h neighboring uninfected tissue condition (r = 0.76, *p* = 1.16 × 10^−6^), enriched in ferroptosis, cysteine and methionine metabolism, and PPAR signaling (Table 5)—suggesting that adjacent uninfected mammary tissue mounts a metabolic and redox-protective response rather than a direct immune response. HMGCL (MM = 0.930, GS = 0.720), the sole overlapping hub gene in this module (Table 1 and Table 2), is consistent with its role in maintaining oxidation-reduction balance and cellular oxidant detoxification in tissues adjacent to the infection site [12,44], positioning it as a candidate regulator of this protective metabolic adaptation. EGFR, identified as the highest-degree hub gene in the 24 hn DEG network (degree = 168; Table 4), substantially higher than its 24 h centrality (degree = 67), suggests its primary role lies in coordinating epithelial recovery and tissue-protective signaling in mammary regions adjacent to the infection site [45], directly consistent with the yellow module’s tissue maintenance pathway enrichment.

### 3.5. Predicted miRNA-Mediated Regulation of Key Hub Genes

Computational miRNA target prediction using miRNet identified putative post-transcriptional regulatory interactions for the identified hub genes (Table 7). Among these, MAPK14 showed the highest predicted miRNA interaction count (*n* = 20), followed by STAT3 (*n* = 18) and GNAI2 (*n* = 16). Certain miRNAs—including bta-miR-1777a/b, bta-miR-2433, and bta-miR-664a—were predicted to target multiple hub genes simultaneously, suggesting broad candidate regulatory coverage across mastitis-associated pathways. All reported miRNA–mRNA associations are sequence-complementarity-based predictions that have not been experimentally validated in the present study and should be interpreted as candidate regulatory interactions requiring confirmation through luciferase reporter assays or miRNA functional studies.

### 3.6. Differential Transcriptional Associations Across Infection Stages and Limitations

The module–trait correlation analysis revealed differential transcriptional associations across infection time points: JUN showed stronger network association with the 6 h post-infection condition within the blue module (r = 0.62, *p* = 2.4 × 10^−4^), while STAT3 was more strongly associated with the 24 h infection condition within the turquoise module (r = 0.86, *p* = 1.07 × 10^−9^), and RAC2 was identified exclusively within the 24 h DEG-derived network (degree = 81, betweenness = 309,020; Table 3). These differential associations suggest potentially distinct transcriptional roles across infection stages; however, they are based on only two post-infection time points and Pearson correlation-based module assignments rather than rigorous time-series modeling, and should therefore be interpreted as stage-associated rather than sequentially causal regulatory patterns.

The mechanistic basis for the upregulation of these hub genes can be contextualized within established *E. coli*-induced signaling cascades. JUN upregulation at 6 h is consistent with rapid TLR4-mediated activation of the MyD88/MAPK pathway by bacterial LPS, leading to c-Jun phosphorylation and AP-1 complex formation—a response occurring within minutes to hours of pathogen recognition [23,34]. STAT3 upregulation at 24 h reflects sustained JAK-STAT signaling downstream of pro-inflammatory cytokines (IL-6, IL-10) released during the innate immune response, wherein JAK1/JAK2 directly phosphorylate STAT3 to drive acute-phase gene expression [24,25]. RAC2 upregulation at 24 h likely reflects the transcriptional signature of infiltrating neutrophils the predominant immune cells recruited to the mammary gland during acute *E. coli* mastitis rather than epithelial cell-intrinsic upregulation, as RAC2 is preferentially expressed in hematopoietic cells and is essential for NADPH oxidase-mediated superoxide production [46,47]. While these mechanistic interpretations are supported by published experimental evidence, the present computational study identifies correlational associations, and establishing direct causality would require gene perturbation experiments in bovine mammary tissue.

Among the three top hub genes, STAT3 and JUN have been experimentally validated in mastitis-related contexts through RT-qPCR in bovine models [23,25,26], lending external experimental confidence to their computational identification. RAC2 emerges as a novel candidate, supported by cross-species functional evidence from murine models [46,47,48], but requires experimental validation in bovine mammary tissue before definitive conclusions can be drawn. The present study is computational in nature, and direct experimental validation through wet lab approaches was beyond the scope of the current work; future studies employing RT-qPCR expression profiling, gene knockdown, and protein-level analyses in bovine mammary tissue are warranted to confirm the functional roles of the identified candidate hub genes. Together, these findings offer a systematic computational characterization of candidate molecular mechanisms underlying *E. coli* mastitis progression in the bovine mammary gland.

## 4. Materials and Methods

### 4.1. Microarray Data Collection and Processing

Microarray gene expression data for *Escherichia coli*-induced bovine mastitis were obtained from Mitterhuemer et al. [49] (GEO accession GSE15025). The study comprised 15 healthy German Holstein-Friesian heifers in mid-lactation, of which ten were experimentally inoculated with *E. coli* and sacrificed at 6 h (*n* = 5) and 24 h (*n* = 5) post-infection, while five served as uninfected controls. Gene expression data were deposited as three independent GEO datasets GSE15019 (6 h infected vs. control; *n* = 10), GSE15020 (24 h infected vs. control; *n* = 10), and GSE15022 (24 h neighboring uninfected tissue vs. control; *n* = 10) (Table 8) yielding 30 microarray samples in total. Total RNA was isolated from mammary gland tissues, converted to biotin-labeled cRNA, and hybridized onto the Affymetrix GeneChip^®^ Bovine Genome Array following standard manufacturer protocols. Raw CEL files were downloaded from GEO, subjected to log_2_ transformation and quantile normalization, and filtered to remove probes with missing values, low signal intensity, or absent gene annotations. Differential expression analysis was performed independently for each dataset using the limma R package version 4.6.0 [50] prior to cross-dataset integration. Inter-dataset batch effects were subsequently corrected using the removeBatchEffect function from the limma R package [50], with each GEO dataset designated as a separate batch, and the batch-corrected expression matrix was used as input for WGCNA co-expression network construction.

### 4.2. Gene Co-Expression Network Construction Using WGCNA

Co-expression network analysis was performed using the WGCNA R package [51,52] applied to the normalized expression profiles of 5000 genes derived from 30 microarray samples across three GEO datasets (GSE15019, GSE15020, GSE15022). Prior to network construction, expression data were normalized and transformed to z-scores to ensure comparability across datasets and minimize technical variability.

A soft-thresholding power (β = 9) was selected based on the scale-free topology criterion (scale-free R^2^ = 0.81, slope = −1.1), ensuring that the resulting network reflected a biologically meaningful scale-free topology commonly observed in biological co-expression networks. The softConnectivity function was applied to assess average connectivity across genes, and hierarchical clustering was performed using the flashClust function. The adjacency matrix was subsequently converted to a topological overlap matrix (TOM) to minimize the effects of spurious correlations, and genes were clustered into modules based on TOM-derived dissimilarity measures. Modules were identified using the cutreeDynamic algorithm (minModuleSize = 30, deepSplit = 2, cut height = 0.25). Modules with highly similar eigengene expression profiles were subsequently merged using a module dissimilarity threshold (MEDissThres) of 0.25 to yield a refined and biologically coherent set of modules. Genes that did not cluster into any specific module were assigned to the gray module and excluded from downstream analyses. It should be noted that the present WGCNA pipeline did not incorporate a pre-filtering step to exclude constitutively expressed housekeeping genes prior to network construction. Genes with stable high-level expression across all conditions such as GAPDH may artificially satisfy downstream filtering thresholds due to broad transcriptional activity rather than infection-specific co-expression patterns, representing a recognized limitation of co-expression network analyses [43]. Accordingly, such genes were excluded from downstream biological interpretation.

### 4.3. Module Eigengene Calculation, Module–Trait Correlation, and Hub Gene Identification

Module eigengenes (MEs) were calculated using the moduleEigengenes() function in the WGCNA R package [51,53], defined as the first principal component of the scaled expression matrix of each module, capturing the dominant axis of expression variation across module members. ME signs were aligned to ensure biological interpretability of correlation directions. Module–trait relationships were assessed via Pearson correlation between MEs and clinical traits (infection status and time post-inoculation: 6 h vs. 24 h), with modules showing |r| > 0.5 and *p* < 0.05 considered significantly correlated [51,52].

Gene-to-module correlation (Module Membership, MM) was calculated as the Pearson correlation between each gene’s expression profile and its assigned module eigengene across all 30 samples (Student’s *t*-test, *p* < 0.05), quantifying intramodular connectivity [51,53]. Gene Significance (GS) was calculated as the Pearson correlation between each gene’s expression profile and the clinical trait of interest, reflecting phenotypic relevance [51]. Candidate genes within significant modules were filtered using MM > 0.80 and GS > 0.50 (*p* < 0.05) for downstream PPI network construction [15,52]. This MM/GS filter served as a preliminary candidate screen final high-confidence hub genes were defined by convergent identification across both WGCNA and PPI analyses.

To confirm that the overlap between WGCNA candidate genes and PPI-derived hub genes was greater than expected by chance, a hypergeometric test was performed [54]

$$P\left(X\geq k\right)=\frac{\sum \left[C\left(K,i\right)\times C\left(N-K,n-i\right)\right]}{C\left(N,n\right)}$$

where *N* is the total number of genes analyzed, K is the number of WGCNA candidate genes, n is the number of PPI-derived hub genes, and k is the observed overlap. Overlaps with *p* < 0.05 were considered statistically significant. Significantly correlated modules were further subjected to GO and KEGG pathway enrichment analyses.

### 4.4. Functional Analysis of Candidate Modules via GO and KEGG

GO and KEGG enrichment analyses were performed for genes in the candidate modules using the OmicShare platform (https://www.omicshare.com/tools) (Accessed on 12 November 2025) [55] with the Bos taurus genome as background. The hypergeometric test with Benjamini–Hochberg FDR correction identified significantly enriched terms (FDR < 0.05, ≥3 genes). Functional annotation covered Biological Process (BP), Molecular Function (MF), and Cellular Component (CC) categories. The top 20 enriched GO terms and KEGG pathways were prioritized based on −log_10_(FDR) values. Results were visualized as bubble plots and network maps.

### 4.5. PPI Network Construction, Module Analysis, and Hub Gene Identification

Biological interactions within the candidate gene modules were analyzed using the STRING v11.0 database (https://string-db.org/) (Accessed on 28 November 2025), which integrates both experimentally validated and predicted protein–protein interactions derived from curated databases, text mining, co-expression, and co-occurrence evidence. The analysis was conducted using the Bos taurus reference proteome, and all duplicate or unannotated gene symbols were excluded prior to network generation to ensure data accuracy. A high-confidence interaction score cutoff of 0.7 (combined score) was applied to filter significant protein–protein interactions [56]. The resulting interaction networks for each module were exported in TSV format from STRING and visualized using Cytoscape v3.10.1 [57]. Network topology parameters—degree, betweenness centrality, closeness centrality, and clustering coefficient—were calculated using the NetworkAnalyzer tool in Cytoscape to evaluate the relative importance of each node in maintaining network connectivity. Subsequently, the cytoHubba plug-in was employed to identify key hub genes based on multiple ranking algorithms, including Degree, Maximal Clique Centrality (MCC), Closeness, and Betweenness [57]. The top 10% of genes ranked by Degree and MCC scores were selected as potential hub candidates. Nodes with degree ≥ 10 or appearing in the top 5% across algorithms were considered core regulatory genes within the PPI network. Network enrichment was evaluated within STRING using Gene Ontology (GO) Biological Process and KEGG pathway annotations, applying a false discovery rate (FDR) < 0.05 as the significance threshold. Finally, the overlapping hub genes identified independently from both the Weighted Gene Co-expression Network Analysis (WGCNA) and PPI-based analysis were designated as “true hub genes,” reflecting their central regulatory roles across both transcriptomic co-expression and protein interaction layers for downstream integrative analysis.

### 4.6. Differential Expression Analysis and DEG Integration

Independent differential expression analyses were conducted on each dataset using the GEO2R analytical platform [58], accessible via the NCBI Gene Expression Omnibus (http://www.ncbi.nlm.nih.gov/geo/geo2r/) (Accessed on 16 December 2025), to ensure analytical consistency across datasets. GEO2R utilizes R-based statistical frameworks to identify genes exhibiting significant differential expression under distinct experimental conditions [59]. The tool internally integrates the GEOquery and limma packages from the Bioconductor project to retrieve raw expression matrices and perform normalization, linear modeling, and hypothesis testing. Prior to analysis, raw microarray intensity values were subjected to log_2_ transformation and quantile normalization to minimize technical variability among samples. Probes with missing values, low signal intensity, or lacking official gene annotations were filtered out to ensure data quality. Each dataset was independently analyzed using the moderated t-statistic approach implemented in limma for robust variance estimation, particularly suitable for small sample sizes. The Benjamini–Hochberg false discovery rate (FDR) correction was applied to adjust *p*-values for multiple testing [60]. Genes were classified as differentially expressed based on an absolute log_2_ fold-change (|log_2_FC|) ≥ 2.0 combined with Benjamini–Hochberg FDR-adjusted *p*-values. For the 24 h and near-24 h (n24 h) infection groups, a standard FDR threshold of <0.05 was applied. For the 6 h infection group, the FDR threshold was relaxed to <0.1, as application of the 0.05 cutoff yielded only less DEGs—insufficient for robust co-expression network construction. This relaxed threshold is consistent with accepted practice in exploratory transcriptomic analyses [61], and the stringent |log_2_FC| ≥ 2.0 filter was retained uniformly across all groups to preserve biological significance.

### 4.7. Validation of Hub Genes Using Machine Learning Classification Model

The identified hub genes were validated using a Support Vector Machine (SVM) classification model with a radial basis function (RBF) kernel (svmRadial), a widely adopted supervised learning algorithm for non-linear biological data classification [62]. The raw gene expression datasets were log_2_-transformed, quantile-normalized, and filtered to retain the expression profiles of the four hub genes across all samples. The data were partitioned into training (70%) and testing (30%) subsets using stratified random sampling to maintain class balance. Model development was implemented using the caret package in R, applying a repeated 5-fold cross-validation (repeated 5 times) strategy to ensure robust model generalization [63]. Hyperparameter tuning was performed using a grid search approach, exploring a cost parameter (C) range of 0.1–10 and a gamma (σ^2^) range of 0.001–1.0 to minimize the cross-validation error and maximize classification performance [64]. The trained SVM model’s predictive performance was assessed on the test set using a confusion matrix to compare the predicted and actual labels. Evaluation metrics included accuracy, sensitivity (recall), specificity, precision, F1 score, and Cohen’s kappa statistic [65]. Additionally, balanced accuracy was used to account for potential class imbalance. Model performance was further visualized through Receiver Operating Characteristic (ROC) curves and Area Under the Curve (AUC) values to quantify discriminative ability. Feature importance scores were extracted using recursive feature elimination (RFE) to confirm the individual contribution of each hub gene to the classifier’s performance. The final SVM model achieving the highest mean cross-validation accuracy and AUC was selected as the optimal model for validating the predictive relevance of the identified hub genes in mastitis response classification.

### 4.8. Identification of miRNAs Targeting Hub Genes

To explore putative post-transcriptional regulatory mechanisms governing the identified hub genes, computational miRNA target prediction was performed using the miRNet 2.0 platform (https://www.mirnet.ca/) (Accessed on 20 December 2025) [66]. This analysis was motivated by evidence that miRNA-mediated regulation represents an important layer of immune gene control during bovine mastitis [17]. The analysis was conducted using Bos taurus as the reference organism, with hub gene symbols uploaded to construct a putative miRNA–gene interaction network. Only interactions with a miRanda confidence score ≥ 0.8 were retained. Network topology parameters including node degree, betweenness centrality (≥0.05), and clustering coefficient were computed to identify candidate regulatory miRNAs. miRNet 2.0 integrates data from miRTarBase, TarBase, miRecords, and miRanda databases [66].

## 5. Conclusions

This study applied a multi-method transcriptomic framework combining WGCNA, differential gene expression analysis, PPI network construction, SVM classification, and computational miRNA target prediction to characterize the host response to *E. coli*-induced bovine mastitis across three experimental conditions. STAT3, JUN, and RAC2 were identified as high-confidence candidate hub genes through convergent evidence across multiple independent analytical frameworks, with JUN showing stronger association with the 6 h infection condition and STAT3 and RAC2 with the 24 h condition. An SVM classifier trained on these hub genes achieved 87.5% accuracy (sensitivity = 100%, specificity = 75%) in distinguishing infected from control samples, supporting their potential as candidate transcriptomic biomarkers. As a purely computational study, all findings require experimental validation in bovine mammary tissue before translational implications can be considered. These results provide a systematic computational foundation for future investigations into the molecular mechanisms underlying *E. coli*-induced bovine mastitis.

## Figures and Tables

**Table 1:** Module membership (MM) and gene significance (GS) of key hub genes in Blue, Turquoise, and Yellow modules.

Node Name	MM	GS
Blue Module		
*BRAF*	0.9196	0.5321
*SNW1*	0.9412	0.6191
*LATS1*	0.9067	0.5213
*LARP4*	0.9605	0.5560
*JUN*	0.8034	0.6206
Turquoise Module		
*GAPDH*	0.8243	0.9500
*STAT1*	0.9470	0.8219
*STAT3*	0.9494	0.7444
*CD44*	0.9371	0.8976
*TP53*	0.8474	0.7215
*MMP9*	0.8006	0.9792
Yellow Module		
*HMGCL*	0.93	0.7200

**Table 2:** Comparison of hub genes identified from WGCNA modules and PPI networks across infection time points.

	Hub Genes in WGCNA	Hub Genes in PPI Network	Hub Genes in PPI Network and Hub Genes in WGCNA
blue module (6 h)	68	14	5
Tourquise (24 h)	1132	6	6
Yellow (24 hn)	67	4	1

**Table 3:** Hub genes from differentially expressed across all three time period (6 h, 24 h, 24 hn).

ID	Name	Degree	Betweenness
327671	RAC2	81	309,020.2
282306	PIK3CA	73	239,669.9
504531	PIK3CD	71	228,471
407217	EGFR	67	261,194.4
509913	WDR46	65	78,102.5
281061	CDK1	62	244,848.6
505846	UTP18	61	57,805.39
515001	NAT10	59	201,044.1
534492	MAPK14	59	177,840.4
535327	MAPK13	58	169,010
539521	GNAI3	58	151,976.5
281791	GNAI2	57	149,525.9
327694	EIF2S1	54	458,957.3
512041	EBNA1BP2	53	90,811.64
521240	WDR75	53	75,859.49

**Table 4:** Final top hub genes identified in both WGNCS and DEG.

WGNCS			DEG		
Node Name	MM	GS	Name	Degree	Betweenness
6 h of hub genes from *WGCNS* and *DEG.*					
BRAF	0.9196	0.5321	FOS	48	3466
SNW1	0.9412	0.6191	LPIN1	20	1520
LATS1	0.9067	0.5213	TRIB3	11	1965.5
LARP4	0.9605	0.556	KLF5	7	435.5
JUN	0.8034	0.6206	NRG1	5	350
24 h of hub genes from *WGNS* and *DEGs*					
GAPDH	0.8243	0.95	RAC2	81	298,039.2
STAT1	0.947	0.8219	PIK3CA	73	183,452.2
STAT3	0.9494	0.7444	PIK3CD	71	173,138.9
CD44	0.9371	0.8976	EGFR	67	242,288.9
TP53	0.8474	0.7215	WDR46	65	71,891.61
MMP9	0.8006	0.9792			
24 h neighbor hub genes from *WGNS* and *DEGs*					
HMGCL	0.93	0.72	EGFR	168	516,017
			SOD2	85	213,555.1
			HSPA8	79	176,484.3
			HSP90AB1	71	243,487.1
			MED10	68	91,429.46

**Table 5:** KEGG pathway enrichment profiles for Blue, Turquoise, and Yellow WGCNA modules showing significantly overrepresented signaling, immune, and metabolic pathways based on FDR-adjusted significance and fold enrichment.

Path	Term	Enrichment FDR	nGenes	Pathway Genes	Fold Enrichment
Blue Module					
bta05211	Renal cell carcinoma	4.40 × 10^−2^	6	70	5.1
bta04012	ErbB signaling pathway	2.40 × 10^−2^	7	82	5.1
bta05223	Non-small cell lung cancer	4.40 × 10^−2^	6	71	5.1
bta04150	mTOR signaling pathway	2.40 × 10^−2^	10	154	3.9
bta04510	Focal adhesion	2.40 × 10^−2^	11	192	3.4
Turquoise Module					
bta05144	Malaria	1.70 × 10^−6^	30	55	2.8
bta05133	Pertussis	3.60 × 10^−6^	35	72	2.5
bta05140	Leishmaniasis	8.10 × 10^−6^	35	75	2.4
bta04066	HIF-1 signaling pathway	1.70 × 10^−5^	43	105	2.1
bta04670	Leukocyte transendothelial migration	1.90 × 10^−5^	45	113	2
Yellow Module					
bta01250	Biosynthesis of nucleotide sugars	4.90 × 10^−2^	4	38	9.1
bta04216	Ferroptosis	4.90 × 10^−2^	4	44	7.9
bta00270	Cysteine and methionine metabolism	4.90 × 10^−2^	4	50	7
bta00520	Amino sugar and nucleotide sugar metabolism	4.90 × 10^−2^	4	50	7
bta03320	PPAR signaling pathway	2.60 × 10^−2^	6	80	6.5

**Table 6:** Performance metrics for microarray analysis using a machine learning model for the hub genes.

Matrices	Values
Accuracy	0.875
Sensitivity	1
Specificity	0.75
Recall	1
F1 score	0.889
Precision	0.8
Kappa	0.75

**Table 7:** Predicted miRNA regulators of key hub genes (STAT3, JUN, and RAC2) and their interaction counts.

Gene	miRNA_Count	miRNAs
STAT3	18	bta-miR-21-5p, bta-miR-320a, bta-miR-10a, bta-miR-17-5p, bta-miR-342, bta-miR-485, bta-miR-551a, bta-miR-1301, bta-miR-2313-5p, bta-miR-2339, bta-miR-2430,
		bta-miR-2432, bta-miR-1777a, bta-miR-1777b, bta-miR-2465, bta-miR-2881, bta-miR-2897, bta-miR-7861
JUN	12	bta-miR-760-5p, bta-miR-940, bta-miR-1281, bta-miR-2327, bta-miR-2328-5p, bta-miR-2387, bta-miR-2433, bta-miR-2449, bta-miR-1777a, bta-miR-2454-5p,
		bta-miR-664a, bta-miR-1247-5p
RAC2	8	bta-miR-432, bta-miR-2305, bta-miR-2309, bta-miR-2327, bta-miR-2428, bta-miR-2433, bta-miR-664a, bta-miR-6517

**Table 8:** Summary of GEO datasets used for time-course transcriptomic analysis of mastitis.

SI. No	Accession No	Time Course	Control	Disease	Total No. of Samples
1	GSE15019	6 h	5	5	10
2	GSE15020	24 h	5	5	10
3	GSE15022	n24 h	5	5	10

## Data Availability

Data is available in NCBI GEO database.

## Supplementary Materials

The following supporting information can be downloaded at https://www.mdpi.com/article/10.3390/ijms27167134/s1.

## Abbreviations

The following abbreviations are used in this manuscript:

WGCNA	weighted gene co-expression network analysis
SVM	Support Vector Machine
DEGs	Differential expressed Genes
GEO	Gene Expression Omnibus
cDNA	complementary DNA
CEL	Cell Intensity File
GS	Gene significance
MM	module membership
TOM	topological overlap matrix
GO	Gene Ontology
KEGG	Kyoto Encyclopedia of Genes and Genomes
FDR	False Discovery Rate
RBF	radial basis function
RFE	recursive feature elimination
PPI	Protein–protein interaction

## References

[1] Hogeveen H., Steeneveld W., Wolf C.A. (2019). Production Diseases Reduce the Efficiency of Dairy Production: A Review of the Results, Methods, and Approaches Regarding the Economics of Mastitis. Annu. Rev. Resour. Econ..

[2] Paramasivam R., Gopal D.R., Dhandapani R., Subbarayalu R., Elangovan M.P., Prabhu B., Veerappan V., Nandheeswaran A., Paramasivam S., Muthupandian S. (2023). Is AMR in Dairy Products a Threat to Human Health? An Updated Review on the Origin, Prevention, Treatment, and Economic Impacts of Subclinical Mastitis. Infect. Drug Resist..

[3] Tommasoni C., Fiore E., Lisuzzo A., Gianesella M. (2023). Mastitis in Dairy Cattle: On-Farm Diagnostics and Future Perspectives. Animals.

[4] Haider A., Ikram M., Shahzadi I., Asif Raza M. (2023). Polymeric Nanoparticles for Bovine Mastitis Treatment.

[5] Kabelitz T., Aubry E., van Vorst K., Amon T., Fulde M. (2021). The Role of *Streptococcus* spp. in Bovine Mastitis. Microorganisms.

[6] Shoaib M., Aqib A.I., Naseer M.A., Bhutta Z.A., Pu W., Tanveer Q., Muzammil I., Kulyar M.F.-E., Younas M.S., Hammad M. (2022). Etiology of Bovine Mastitis. Mastitis in Dairy Cattle, Sheep and Goats.

[7] Cobirka M., Tancin V., Slama P. (2020). Epidemiology and Classification of Mastitis. Animals.

[8] Hoque M.N., Istiaq A., Clement R.A., Sultana M., Crandall K.A., Siddiki A.Z., Hossain M.A. (2019). Metagenomic deep sequencing reveals association of microbiome signature with functional biases in bovine mastitis. Sci. Rep..

[9] Aavash K., Sajita G., Narayan G.C., Kumar S.A. (2023). Prevalence of Subclinical Mastitis and Antibiogram of *Escherichia coli* in Cow Milk of Western Chitwan. J. Vet. Med. Res..

[10] Blum S.E., Heller E.D., Sela S., Elad D., Edery N., Leitner G. (2015). Genomic and Phenomic Study of Mammary Pathogenic *Escherichia coli*. PLoS ONE.

[11] Tong X., Barkema H.W., Nobrega D.B., Xu C., Han B., Zhang C., Yang J., Li X., Gao J. (2025). Virulence of Bacteria Causing Mastitis in Dairy Cows: A Literature Review. Microorganisms.

[12] Ghahramani N., Shodja J., Rafat S.A., Panahi B., Hasanpur K. (2021). Integrative Systems Biology Analysis Elucidates Mastitis Disease Underlying Functional Modules in Dairy Cattle. Front. Genet..

[13] Younis S., Javed Q., Blumenberg M. (2016). Meta-Analysis of Transcriptional Responses to Mastitis-Causing *Escherichia coli*. PLoS ONE.

[14] Naserkheil M., Ghafouri F., Zakizadeh S., Pirany N., Manzari Z., Ghorbani S., Banabazi M.H., Bakhtiarizadeh M.R., Huq A., Na Park M. (2022). Multi-Omics Integration and Network Analysis Reveal Potential Hub Genes and Genetic Mechanisms Regulating Bovine Mastitis. Curr. Issues Mol. Biol..

[15] Bakhtiarizadeh M.R., Mirzaei S., Norouzi M., Sheybani N., Vafaei Sadi M.S. (2020). Identification of Gene Modules and Hub Genes Involved in Mastitis Development Using a Systems Biology Approach. Front. Genet..

[16] Wang X., Xiu L., Hu Q., Cui X., Liu B., Tao L., Wang T., Wu J., Chen Y. (2013). Deep Sequencing-Based Transcriptional Analysis of Bovine Mammary Epithelial Cells Gene Expression in Response to In Vitro Infection with *Staphylococcus aureus* Stains. PLoS ONE.

[17] Hasankhani A., Bakherad M., Bahrami A., Shahrbabak H.M., Pecho R.D.C., Shahrbabak M.M. (2023). Integrated analysis of inflammatory mRNAs, miRNAs, and lncRNAs elucidates the molecular interactome behind bovine mastitis. Sci. Rep..

[18] Goulart D.B., Mellata M. (2022). *Escherichia coli* Mastitis in Dairy Cattle: Etiology, Diagnosis, and Treatment Challenges. Front. Microbiol..

[19] Khan M.Z., Khan A., Xiao J., Ma J., Ma Y., Chen T., Shao D., Cao Z. (2020). Overview of Research Development on the Role of NF-κB Signaling in Mastitis. Animals.

[20] Dorrington M.G., Fraser I.D.C. (2019). NF-κB Signaling in Macrophages: Dynamics, Crosstalk, and Signal Integration. Front. Immunol..

[21] Chen Z., Chu S., Wang X., Sun Y., Xu T., Mao Y., Loor J.J., Yang Z. (2019). MiR-16a Regulates Milk Fat Metabolism by Targeting Large Tumor Suppressor Kinase 1 (LATS1) in Bovine Mammary Epithelial Cells. J. Agric. Food Chem..

[22] Zhou C., Li C., Cai W., Liu S., Yin H., Shi S., Zhang Q., Zhang S. (2019). Genome-wide association study for milk protein composition traits in a Chinese Holstein population using a single-step approach. Front. Genet..

[23] Bi Y., Ding Y., Wu J., Miao Z., Wang J., Wang F. (2020). *Staphylococcus aureus* induces mammary gland fibrosis through activating the TLR/NF-κB and TLR/AP-1 signaling pathways in mice. Microb. Pathog..

[24] Khan M.Z., Khan A., Xiao J., Ma Y., Ma J., Gao J., Cao Z. (2020). Role of the JAK-STAT Pathway in Bovine Mastitis and Milk Production. Animals.

[25] Zhou J., Lian S., Geng Z., Yang Y., Wu R., Wang J. (2025). *Lactiplantibacillus plantarum* promotes lactoferrin synthesis and secretion in bovine mammary epithelial cells through STAT3 and AP-1 transcription factor pathways. Vitr. Cell. Dev. Biol. Anim..

[26] Piantoni P., Wang P., Drackley J.K., Hurley W.L., Loor J.J. (2010). Expression of Metabolic, Tissue Remodeling, Oxidative Stress, and Inflammatory Pathways in Mammary Tissue during Involution in Lactating Dairy Cows. Bioinform. Biol. Insights.

[27] Alhussien M.N., Panda B.S.K., Dang A.K. (2021). A Comparative Study on Changes in Total and Differential Milk Cell Counts, Activity, and Expression of Milk Phagocytes of Healthy and Mastitic Indigenous Sahiwal Cows. Front. Vet. Sci..

[28] Gonen E., Nedvetzki S., Naor D., Shpigel N.Y. (2008). CD44 is highly expressed on milk neutrophils in bovine mastitis and plays a role in their adhesion to matrix and mammary epithelium. Vet. Res..

[29] Liapi M., Botsaris G., Arsenoglou C., Markantonis N., Michael C., Antoniou A., Pipis C. (2021). Rapid Detection of *Mycoplasma bovis*, *Staphylococcus aureus* and *Streptococcus agalactiae* in Cattle Bulk Tank Milk in Cyprus and Relations with Somatic Cell Counts. Pathogens.

[30] Santos A.A., Lopes C.C., Marques R.M., Amorim I.F., Gärtner M.F., de Matos A.J.F. (2012). Matrix metalloproteinase-9 expression in mammary gland tumors in dogs and its relationship with prognostic factors and patient outcome. Am. J. Vet. Res..

[31] Chattopadhyay M., Jenkins E.C., Janssen W., Mashaka T., Germain D. (2025). Differential ER stress responses during lactation and postpartum outcomes in mice depending on their mitochondrial genotype. Nat. Commun..

[32] Zhang Z., Yao Y., Yang J., Jiang H., Meng Y., Cao W., Zhou F., Wang K., Yang Z., Yang C. (2023). Assessment of adaptive immune responses of dairy cows with *Burkholderia contaminans*-induced mastitis. Front. Microbiol..

[33] Sharifi S., Pakdel A., Ebrahimie E., Aryan Y., Ghaderi Zefrehee M., Reecy J.M. (2019). Prediction of key regulators and downstream targets of *E. coli* induced mastitis. J. Appl. Genet..

[34] Zhang K., Zhang R., Zhang Y., Zhang M., Su H., Zhao F., Wang D., Cao G., Zhang Y. (2025). Regulation of LPS-Induced Inflammatory Responses in Bovine Mammary Epithelial Cells via TLR4-Mediated NF-κB and MAPK Signaling Pathways by Lactoferrin. Life.

[35] Wu J., Li L., Sun Y., Huang S., Tang J., Yu P., Wang G. (2015). Altered Molecular Expression of the TLR4/NF-κB Signaling Pathway in Mammary Tissue of Chinese Holstein Cattle with Mastitis. PLoS ONE.

[36] Yan S., Ju X., Lao J., Wen Z., Yong Y., Li Y., Li Y. (2024). Overexpression of the Mas1 gene mitigated LPS-induced inflammatory injury in mammary epithelial cells by inhibiting the NF-κB/MAPKs signaling pathways. Front. Vet. Sci..

[37] de Lima A.O., Koltes J.E., Diniz W.J.S., de Oliveira P.S.N., Cesar A.S.M., Tizioto P.C., Afonso J., de Souza M.M., Petrini J., Rocha M.I.P. (2020). Potential Biomarkers for Feed Efficiency-Related Traits in Nelore Cattle Identified by Co-expression Network and Integrative Genomics Analyses. Front. Genet..

[38] Xing Y., Tang Y., Chen Q., Chen S., Li W., Mi S., Yu Y. (2024). The role of RNA epigenetic modification-related genes in the immune response of cattle to mastitis induced by *Staphylococcus aureus*. Anim. Biosci..

[39] Cao Y., Hu G., Li W., Wang J., Ge Y., Li F., Guo W., Kan X., Fu S., Liu J. (2022). Lysine promotes proliferation and β-casein synthesis through the SLC6A14-ERK1/2-CDK1-mTOR signaling pathway in bovine primary mammary epithelial cells. J. Therm. Biol..

[40] Essa B., Al-Sharif M., Abdo M., Fericean L., Ateya A. (2023). New Insights on Nucleotide Sequence Variants and mRNA Levels of Candidate Genes Assessing Resistance/Susceptibility to Mastitis in Holstein and Montbéliarde Dairy Cows. Vet. Sci..

[41] Henning D., Valdez B.C. (2001). Expression of p40/Epstein–Barr Virus Nuclear Antigen 1 Binding Protein 2. Biochem. Biophys. Res. Commun..

[42] Lee L., Whittall J.B. (2025). WDR75: An essential protein for ribosome assembly undergoing purifying selection. PLoS ONE.

[43] Rhoads R.P., McManaman C., Ingvartsen K.L., Boisclair Y.R. (2003). The Housekeeping Genes GAPDH and Cyclophilin Are Regulated by Metabolic State in the Liver of Dairy Cows. J. Dairy Sci..

[44] Darang E., Pezeshkian Z., Mirhoseini S.Z., Ghovvati S. (2023). Identification of Key Genes and Potential Pathways Associated with Mastitis Induced by *E. coli*. Biochem. Genet..

[45] Morato A., Martignani E., Miretti S., Baratta M., Accornero P. (2021). External and internal EGFR-activating signals drive mammary epithelial cells proliferation and viability. Mol. Cell. Endocrinol..

[46] Gu Y., Byrne M.C., Paranavitana N.C., Aronow B., Siefring J.E., D’SOuza M., Horton H.F., Quilliam L.A., Williams D.A. (2002). Rac2, a Hematopoiesis-Specific Rho GTPase, Specifically Regulates Mast Cell Protease Gene Expression in Bone Marrow-Derived Mast Cells. Mol. Cell. Biol..

[47] Yang F.-C., Kapur R., King A.J., Tao W., Kim C., Borneo J., Breese R., Marshall M., Dinauer M.C., A Williams D. (2000). Rac2 Stimulates Akt Activation Affecting BAD/Bcl-XL Expression while Mediating Survival and Actin Function in Primary Mast Cells. Immunity.

[48] Yu H., Leitenberg D., Li B., Flavell R.A. (2001). Deficiency of Small GTPase Rac2 Affects T Cell Activation. J. Exp. Med..

[49] Mitterhuemer S., Petzl W., Krebs S., Mehne D., Klanner A., Wolf E., Zerbe H., Blum H. (2010). *Escherichia coli* infection induces distinct local and systemic transcriptome responses in the mammary gland. BMC Genom..

[50] Ritchie M.E., Phipson B., Wu D., Hu Y., Law C.W., Shi W., Smyth G.K. (2015). limma powers differential expression analyses for RNA-sequencing and microarray studies. Nucleic Acids Res..

[51] Langfelder P., Horvath S. (2008). WGCNA: An R package for weighted correlation network analysis. BMC Bioinform..

[52] Liao Y., Wang Y., Cheng M., Huang C., Fan X. (2020). Weighted Gene Coexpression Network Analysis of Features That Control Cancer Stem Cells Reveals Prognostic Biomarkers in Lung Adenocarcinoma. Front. Genet..

[53] Zhang B., Horvath S. (2005). A general framework for weighted gene co-expression network analysis. Stat. Appl. Genet. Mol. Biol..

[54] Rivals I., Personnaz L., Taing L., Potier M.C. (2007). Enrichment or depletion of a GO category within a class of genes: Which test?. Bioinformatics.

[55] Muley V.Y. (2025). Functional Insights Through Gene Ontology, Disease Ontology, and KEGG Pathway Enrichment. Computational Virology.

[56] Szklarczyk D., Gable A.L., Lyon D., Junge A., Wyder S., Huerta-Cepas J., Simonovic M., Doncheva N.T., Morris J.H., Bork P. (2019). STRING v11: Protein–protein association networks with increased coverage, supporting functional discovery in genome-wide experimental datasets. Nucleic Acids Res..

[57] Su G., Morris J.H., Demchak B., Bader G.D. (2014). Biological Network Exploration with Cytoscape 3. Curr. Protoc. Bioinform..

[58] Kalyan G.U., Pranathi Reddy D., Chandrakanth G., Pooja B., Anitha V., Vivek D. (2023). Gene Association Disease Prediction by GEO2R Tool. 2023 International Conference on Evolutionary Algorithms and Soft Computing Techniques (EASCT).

[59] Song Y.J., Li G., He J.H., Guo Y., Yang L. (2015). Bioinformatics-Based Identification of MicroRNA-Regulated and Rheumatoid Arthritis-Associated Genes. PLoS ONE.

[60] Kontou P.I., Pavlopoulou A., Bagos P.G. (2018). Methods of Analysis and Meta-Analysis for Identifying Differentially Expressed Genes. Genetic Epidemiology: Methods and Protocols.

[61] Benjamini Y., Hochberg Y. (1995). Controlling the False Discovery Rate: A Practical and Powerful Approach to Multiple Testing. J. R. Stat. Soc. Ser. B Methodol..

[62] Huang K.K., Zheng H.L., Li S., Zeng Z.Y. (2022). Identification of hub genes and their correlation with immune infiltration in coronary artery disease through bioinformatics and machine learning methods. J. Thorac. Dis..

[63] Kuhn M. (2008). Building Predictive Models in R Using the caret Package. J. Stat. Softw..

[64] James G., Witten D., Hastie T., Tibshirani R. (2021). An Introduction to Statistical Learning.

[65] Guyon I., Weston J., Barnhill S., Vapnik V. (2002). Gene Selection for Cancer Classification using Support Vector Machines. Mach. Learn..

[66] Chang L., Zhou G., Soufan O., Xia J. (2020). miRNet 2.0: Network-based visual analytics for miRNA functional analysis and systems biology. Nucleic Acids Res..

