# Supplementary material for: Multi-Layer Transcriptome Analysis Uncovers Molecular Signatures of Coliform Mastitis in Lactating Cows
Source: Int J Mol Sci. 2026 Aug 9;27(16):7134. doi: 10.3390/ijms27167134 (PMC13513401; doi:10.3390/ijms27167134)
Supplement: Supplementary file 1 [file ijms-27-07134-s001.zip › Supplementary_Table S1_word.pdf]

Table S1. Module-wise distribution of genes identified through co-expression network analysis.

Blue_Module	Turquoise_Module	Magenta_Module
IL33	S100A9	AKR1C3
ZBTB41	HP	H4C2
VL1X	S100A12	THRSP
CREB3L2	S100A8	STMN4
GLG1	SDS	ASB11
PMEPA1	CXCL2	LOC100296421
MOSMO	CFB	HES6
TTC3	SAA3	RNASE12
SZRD1	LOC508459	LOC100297399
PHF3	CXCL8	FGR
KLF6	CXCR1	H2AC6
MTPN	SLC6A14	NCAPH
MINDY2	DEFB4A	PPM1K
SLC39A10	SAA2	CD14
JUN	PRSS2	CITED4
IQGAP2	GRO1	ZMYND12
FGD4	CHI3L1	ACYP2
PDXK	CXCL5	SH3RF2
SLC38A2	PTX3	PPP1R3C

BLTP3B	IL1RN	TMEM154
PHF6	ADGRE3	ECM1
MCOLN3	RUBCNL	SLC39A14
ARMC1	LOC516849	RIMS4
PTPRK	LBP	TAFA1
ATP6V1A	PLEK	KRT19
TBC1D5	LOC785756	RCAN2
HMGCS1	PHYHIPL	GLRX5
PRRG4	DEFB7	SPNS2
CCNT1	C3H1orf162	SLC6A20
ARIH1	LPL	MTARC1
TMEM47	NFE2	GJB5
CEBPG	RBP1	ATF4
AKAP1	IL1R2	HMGCR
HYCC2	CD55	SEMA4A
BCLAF1	CCR1	LCP2
FERMT2	SHISAL2B	PDIA6
AGPAT5	TECTB	STX19
NAV3	SLC11A1	CDH2
UBR1	LAP	LOC104972526
DYNC1LI2	TNFRSF6B	EMC6

PEF1	MT1A	CDC42SE1
LARP1	IL18BP	FBLIM1
ZBTB43	MT2A	
ROCK1	TREM1	
PNPO	C4BPA	
SNW1	LOC100847724	
ANKRD10	OLR1	
ZNF106	PRSS22	
GHR	C10H15orf48	
FNDC3B	LOC783399	
INO80	VNN2	
PDPK1	SLC34A2	
ERAP2	LOC100296618	
UBE2B	LOC407171	
ROCK2	PTAFR	
NAA25	LOC613358	
THBS1	FCER1G	
ZNF24	HPGD	
SH3PXD2A	RETN	
PAPOLB	NCF1	
LOC506495	CP	

TCF4	TNC	
DLC1	IGSF6	
LARP4	CCL2	
BRWD1	OLFM4	
LOC515150	SOCS3	
STAM2	RIPOR2	
RBPJ	CD68	
LOC100848689	PSTPIP2	
RLIM	BAX	
TXLNG	SCNN1G	
SPEF1	S100A2	
RUFY3	LYZ	
PRMT9	LOC100126815	
HSP90AA1	ARG2	
EHD4	ATP13A4	
TESK2	CCL19	
LATS1	ITGAM	
TNRC6A	HCK	
EFR3A	CEBPB	
ITGA8	TACSTD2	
LOC510377	TGM3	

TEAD1	CFI	
TNPO1	BoLA	
SLC7A1	CCDC184	
BAG4	PTHLH	
HIVEP2	NCF4	
TSC1	SELL	
CBLB	SRGN	
MYLK	SAMSN1	
ADGRL2	ACSL6	
LOC786352	SHISA3	
HABP4	TMEM156	
PIK3R1	SPADH1	
BAZ1A	FZD5	
PEX10	KERA	
METTL2A	SPI1	
BMP2K	CCL26	
KMT2C	BCL2A1	
STAB1	RND1	
CCAR2	SLC1A1	
SIPA1L1	HMOX1	
ALG13	CD69	

MAGI3	LOC507615	
PABPN1	SLA	
SHOC2	IL6	
KDSR	CD48	
NBN	CTSC	
FAM117A	SOD2	
ARMH4	ISG15	
OXR1	C5H12orf75	
YTHDC2	FCN1	
SPAG16	FRRS1	
TIMD4	CAPN6	
KHDRBS1	VCAN	
LOC783879	CIDEA	
RBFOX2	CD53	
CGGBP1	CD44	
GCFC2	LDHA	
TRIO	ALOX5AP	
GRB10	NEB	
MTF2	MPZL2	
LURAP1L	LOC513055	
NAGK	SLC16A3	

IREB2	TNFAIP6	
STK4	SCIN	
SETD5	LCP1	
APOD	SLC30A2	
JCAD	MAP3K8	
CCDC17	RASL11B	
SCNN1A	GPX3	
EFNA5	PYCARD	
SPATS2L	MYBPC1	
FBXL3	FCAR	
LGALS4	LOC789748	
CD2AP	THEMIS2	
PIGQ	LOC788634	
EIF2A	ST6GALNAC3	
SLTM	RAB15	
COQ4	GNLY	
CCN2	SNX10	
LBH	P2RY13	
ORAI1	SAMD9	
SLC15A2	CHI3L2	
PIGP	CA6	

AMDHD1	ACSM1	
TAOK3	STATH	
CLTC	ZBTB16	
TTYH3	LOC782977	
KDM2A	CXCL13	
EIF2S3	SDC4	
DCAF1	PTI	
ZYG11B	BACE2	
CLEC14A	ADGRF1	
BRWD3	IRAK1	
RAB4B	PDK4	
LZTS2	HCLS1	
RRAGD	CCL16	
KRAS	AMPD2	
TAF3	LOC618416	
SMARCA4	CCL4	
UNC50	ITGB2	
AGO3	RYR1	
LOC534630	TFF3	
VWF	ARPC3	
PDE12	LOC104975663	

ATG4A	F5	
CNGA3	SLC39A2	
IRAK1BP1	CDH16	
RHBG	XAF1	
IL2RB	ADGRE5	
AMOTL1	BASP1	
KDM4B	ZBTB44	
SRM	CTSH	
PTPN1	MX1	
PLXND1	C5AR2	
TMEM123	CLDN1	
ZDHHC14	RYR3	
ABRAXAS2	GALM	
RGS12	FBXO33	
PACC1	PABPC1L	
SLC25A16	PLAC8B	
SBNO1	NFKB2	
RBM25	SLC39A12	
FSD1L	SLC26A9	
TSPAN4	LST1	
PGAP1	NFAM1	

CCDC138	NARF	
BRAF	MS4A8	
TNFRSF19	GPAM	
ADCK5	TGFBI	
LOC510193	STAT3	
ZNF532	TPPP3	
TUSC1	H4C14	
EIF4G3	SLC38A7	
ATXN7L1	LOC508666	
TINAGL1	WDFY4	
FBXO9	ANGPT2	
CLINT1	PDPN	
EXOC5	MAPK4	
WWC2	LAT2	
ENY2	PTPRE	
ZFAND2A	HHEX	
RTP4	MXD1	
APC	UGDH	
KDM6B	NR4A3	
ST8SIA4	RBM20	
DUSP1	FKBP5	

PCYOX1	MEDAG	
HPN	SERPINA1	
ARFGEF3	LTA4H	
FAM222B	CASP4	
PRPF40A	NT5C2	
HEPH	PDE4B	
ANKRD52	USF1	
ACAP1	TGIF1	
TOM1L2	RAB7B	
PTGES3L	SLCO4A1	
MAP7D3	NR3C2	
TMSB4X	PIGT	
KIF3A	TLR2	
H1-0	TC2N	
DCAF8	PILRA	
GSN	SNX24	
CLIC4	MCTP2	
STAT5B	CCL20	
VRK3	ISM1	
COL16A1	PGM5	
PHC1	ACSL5	

IGF2R	ADAMTS4	
PROZ	CYP11A1	
D2HGDH	LOC504861	
TSTD2	ALDH5A1	
ZNF205	EVA1C	
LOC100847995	LOC100298356	
CCDC85C	C3	
TMEM41B	SLC7A5	
SLC25A1	ZC3H12A	
GPX2	CDK6	
KANSL1	C2CD4B	
ACKR4	SERPINE1	
YPEL1	ANXA8L1	
LOC100848703	DNAJC12	
CPEB1	BDH1	
SNTB2	CXADR	
CCDC136	DAGLB	
CEP43	SREK1IP1	
RNPEP	ADORA2B	
MITD1	MYO1F	
ASAP1	ETS2	

GGT5	ERBB3	
OPLAH	JUNB	
CCAR1	TMEM120B	
NFIC	FOLR1	
PURG	PARP9	
MFSD6	MSTN	
MMP1	CXCR4	
ERRFI1	IFNAR1	
PHYH	TMEM51	
TPK1	OAS1Y	
CAMK2N1	IKZF2	
AGFG2	EMB	
MTMR11	LOC100139670	
PMF1	CEBPD	
PCDH17	LRATD1	
MKS1	THADA	
ECH1	QNG1	
NFAT5	LIPA	
GPR137B	RFFL	
NR2F2	BAG3	
PRPF39	MAOA	

CCDC115	TRIB1	
SKAP1	LAT	
ARF5	MEFV	
DDX42	SLC2A3	
STRADB	IER5	
PRR5	NFKBIA	
TPGS1	SLC7A7	
SPRYD4	ME2	
SRSF6	AQP9	
ZNF318	RHOG	
AP1M2	SULT1B1	
MXI1	CAPN14	
HYOU1	SELENBP1	
ADAM17	MKNK1	
C16H1orf115	FABP4	
ZNF605	LRRC8D	
HDLBP	RNF121	
GRAMD1A	RAP1GAP2	
UBR3	IL1RAP	
LRRN2	MCM6	
HNRNPA2B1	CLIC1	

ACOT4	COLEC12	
MISP3	GNB4	
BRD3	KLF12	
HOOK2	ABLIM1	
CFAP418	H19	
AFG3L2	IL18	
NEURL4	IL13RA1	
CNOT9	SLC7A8	
ZNF48	RNF183	
ATF7	RCC2	
PRRG1	CYP4B1	
PRLR	SEMA3E	
MED19	ADAM19	
ZNF543	ANG	
HOMER1	EGR1	
AOC1	UPP1	
TAX1BP1	LOC101905672	
CETN2	MUC15	
GOLPH3	CYRIB	
TMEM35A	ADAMTSL4	
GGNBP2	RGS19	

TNFRSF1B	ARRDC4	
THRA	PARP12	
CTSF	QSOX1	
MYG1	KLHL28	
HPS6	C15H11orf34	
MYO1C	TSC22D3	
TTN	GSAP	
MAP4K2	NAALADL2	
DCUN1D2	TNIP1	
TNRC6B	VCAM1	
MCCC1	FERMT3	
OAZ2	LAPTM5	
EPHA2	LOC282340	
RSKR	KCNK5	
PPP1R2	ACSL4	
PTPN11	APBB1IP	
CCNYL1	SLC22A3	
TMEM63A	LOC507550	
FOXP2	BTNL9	
ETV4	CD34	
SCHIP1	TSPO	

SMCHD1	RBMS3	
AJUBA	S100A14	
IP6K1	SP100	
EXOC8	SQOR	
SPATA2	CSGALNACT2	
IQANK1	PEBP4	
ATF6	HRH1	
PMPCB	PIM1	
EEF1AKMT3	LOC617565	
SLC29A2	NT5E	
YPEL5	MARCHF3	
PAK6	TTC36	
POMT1	A2M	
HSBP1	EHBP1L1	
MCC	DSE	
DUSP7	ACACA	
CTSV	TMEM106A	
EIF3G	RAC2	
SERPINA11	FASN	
NEMP2	FES	
RGMB	TGM2	

RHOBTB1	VWA1	
KIAA2026	CCR5	
VAMP4	RASGEF1B	
TRMT61B	LOC100336669	
MIR568	MIPEP	
ZDHHC20	CLDN11	
TOMM5	FGF2	
IQCE	CLMN	
MRM2	GEM	
ERLIN1	CH25H	
JADE1	RRAD	
TST	SULT1E1	
	C15H11orf96	
	SNN	
	ARF2	
	IDO1	
	ZFP36	
	SPINK4	
	LMNB1	
	OXTR	
	FOS	

	IGF2	
	SHCBP1	
	RGS1	
	UCK2	
	CD5L	
	CPOX	
	DKK1	
	BIRC3	
	ST6GAL1	
	SH3KBP1	
	PCLAF	
	RASGRP3	
	SLC5A5	
	ICAM1	
	MAGI1	
	VCL	
	GPR27	
	CCN1	
	ALDH1A3	
	AQP3	
	E2F2	

	SYT17	
	SEC16B	
	SLC35F6	
	VMP1	
	IL6R	
	GPD1	
	PLEKHO2	
	IL10RB	
	ATF3	
	SLC16A6	
	PLAU	
	ABCA1	
	ZNF217	
	PDGFRL	
	EBD	
	NAIP	
	MARCKSL1	
	GLT8D2	
	SH3RF1	
	HAS2	
	BOLA-DQA5	

	GSR	
	PDE3B	
	PFKFB3	
	CNGA1	
	MMP9	
	RHPN2	
	BTN1A1	
	ARL4D	
	SERPINE2	
	LSM14B	
	FAR1	
	SDC2	
	LOC504773	
	FGL2	
	SLC16A12	
	LOC337917	
	CHAC1	
	SRD5A1	
	CUTAL	
	NPL	
	LOC504548	

	MID1	
	GRAMD2A	
	IER3	
	SGK1	
	TXNL1	
	CD244	
	THEM4	
	ZPR1	
	FGL1	
	CASP1	
	PARP14	
	MED20	
	KLK10	
	E2F3	
	CA2	
	SCRN3	
	ENTPD3	
	EZR	
	TMEM176B	
	PPP2R3C	
	LOC100336589	

	LGALS3	
	TNFRSF1A	
	RASA2	
	MFSD2A	
	KLHL8	
	RPS6KA1	
	MTHFD1L	
	LOC509283	
	GPAT3	
	GBP1	
	TEX12	
	TGFB2	
	TMX1	
	RASSF4	
	RBM43	
	AAK1	
	SLC9A7	
	FAM114A1	
	ENPP3	
	MCM4	
	ARHGAP42	

	PMAIP1	
	PSMB8	
	CARM1	
	CYB561	
	TNIK	
	CYBA	
	LRRC17	
	RSAD2	
	BTBD3	
	CDC37L1	
	SNF8	
	ACKR1	
	MELK	
	TRIM36	
	FYN	
	CCDC80	
	ACY1	
	MMRN1	
	SDHAF4	
	DOK6	
	MID1IP1	

	PTGS2	
	GPX1	
	PISD	
	C18H16orf87	
	PARP3	
	IGFBP4	
	CD36	
	BOLA	
	MX2	
	IPMK	
	STRAP	
	IL2RG	
	NFKBIE	
	LOC404103	
	ADA	
	SPON1	
	KRT8	
	NUPR2	
	GMIP	
	TAPBPL	
	PBX2	

	PARVG	
	BICC1	
	PTPRC	
	LOC509034	
	STK24	
	PRKCB	
	RAC3	
	TMPO	
	EFHC1	
	AP1S2	
	MXRA5	
	MOCOS	
	GFPT2	
	F3	
	CSF3	
	ALPL	
	HCAR2	
	CNN2	
	CKAP4	
	ZWINT	
	CLEC6A	

	ANGPTL4	
	IQGAP1	
	PLA2G3	
	RETREG1	
	ASZ1	
	RUNX2	
	TAP1	
	BPIFB1	
	IRF9	
	EMP3	
	RANBP10	
	LOC101908206	
	MPC2	
	CTSA	
	PLXNC1	
	ZC3HAV1	
	PCGF6	
	ASRGL1	
	ACE2	
	CSF2RA	
	NQO1	

	FBXO32	
	LPIN1	
	UBD	
	JAML	
	PDCD6IP	
	AHCY	
	STEAP3	
	HNMT	
	IL1B	
	OSBPL11	
	WLS	
	TUBB	
	IFI16	
	CYBB	
	RAB24	
	OPTN	
	NR4A1	
	IFIH1	
	FAM110C	
	KCTD12	
	SOAT1	

	GLUL	
	COBLL1	
	NCF2	
	WTAP	
	PABPC4	
	KCNMB4	
	TUBB2B	
	EDN1	
	CALCRL	
	AGMO	
	RPS6KA3	
	BANK1	
	DRAM2	
	ABCB7	
	SLC25A24	
	ACAD10	
	CLOCK	
	BRD9	
	SLC4A3	
	TPST2	
	HMGB3	

	COPA	
	DNAJB1	
	RAB20	
	CDK7	
	GEMIN2	
	APP	
	MMP23	
	SSR3	
	ERCC5	
	PKM	
	AGO4	
	TBC1D14	
	ELOVL6	
	SUSD3	
	STX2	
	SAFB2	
	KLF5	
	SERPINB8	
	KRT14	
	GPD2	
	ARHGAP45	

	LOC504730	
	IL10RA	
	RBP4	
	HEXB	
	CCDC170	
	CFP	
	ASL	
	DAPP1	
	BRI3	
	HSPA1A	
	ATP9A	
	UNG	
	SH3TC2	
	SRXN1	
	UBN2	
	GK	
	RAB27A	
	RCAN3	
	NAP1L5	
	MCOLN2	
	PSTPIP1	

	IFI44	
	AAAS	
	B4GALT1	
	HMGB2	
	SLC5A9	
	RASD1	
	ERMP1	
	ACSS3	
	STOM	
	HAUS1	
	GPSM2	
	MN1	
	GPHN	
	EGFR	
	APOBEC3Z1	
	GNPDA1	
	FOXA1	
	DIS3	
	CDK12	
	TMEM176A	
	LY86	

	ARHGAP15	
	TMEM35B	
	RHNO1	
	DOCK7	
	LOC101902029	
	FAM76B	
	CTSZ	
	ZDHHC2	
	SLC25A32	
	MAP2K3	
	LOC100847120	
	HMGB1	
	DES	
	MB21D2	
	H1-10	
	TANGO2	
	BAZ2A	
	C1QTNF5	
	ZNF281	
	CTSK	
	FEM1C	

	TLE1	
	GCNT4	
	CD109	
	ANXA1	
	CCNG2	
	PFN1	
	FABP3	
	ITGB6	
	CKS2	
	ABCD3	
	SORBS1	
	LETMD1	
	DENND4A	
	ARMCX6	
	PSEN1	
	PDLIM4	
	AOX1	
	TLR3	
	SASH1	
	BTK	
	GUCY1A1	

	PLEKHA5	
	KRT10	
	TTC33	
	CPD	
	FUT6	
	RNASET2	
	SERPINB1	
	SRP72	
	PRR13	
	FRK	
	TAPT1	
	SSX2IP	
	LGMN	
	CRLF3	
	AREG	
	ENO4	
	WRAP53	
	MRGBP	
	SERTAD4	
	YPEL3	
	TMEM70	

	TSPAN19	
	DST	
	RAMAC	
	DNAJC6	
	PSEN2	
	MINDY1	
	ST3GAL6	
	DFFA	
	GSE1	
	SMAD9	
	MCMBP	
	MOSPD1	
	CXCL14	
	LOC523126	
	NUSAP1	
	TSPAN13	
	PCDH7	
	ASAH1	
	AATF	
	IFNAR2	
	CASP7	

	TATDN2	
	PRR15L	
	RASSF9	
	RBM3	
	MPC1	
	GJA1	
	LAMA2	
	ANK2	
	DCLK1	
	MICALL2	
	LOC100848596	
	KCNMA1	
	DOCK5	
	HLF	
	SERF1A	
	SAFB	
	ADGRF5	
	CORO1A	
	ENTPD1	
	SLC16A1	
	INSIG1	

	ZNF705A	
	H2BC4	
	MCL1	
	LGALS3BP	
	BIN2	
	RILPL2	
	ADSS2	
	SEMA4D	
	PIK3AP1	
	LRRC8E	
	ARHGAP30	
	PITHD1	
	RIDA	
	ICAM3	
	WDR1	
	GCLC	
	TMBIM1	
	CATHL5	
	CCDC69	
	ECT2	
	DTWD2	

	IFI30	
	HOMER2	
	FN1	
	EFHD2	
	EVI2B	
	MPHOSPH8	
	RASEF	
	AMPD3	
	DHRS3	
	ADAM12	
	AOX4	
	HAPLN1	
	CILP	
	DELE1	
	GFRA2	
	TRAF3IP2	
	CTSS	
	TJP3	
	FXYD2	
	PDSS1	
	IFI6	

	MAP3K21	
	CCN3	
	LOC513969	
	SERPINA3-1	
	GABRB3	
	ANXA2	
	CCDC88A	
	NUDT17	
	LTF	
	AP4S1	
	SMOC1	
	SLC31A2	
	MAU2	
	EID3	
	TTC17	
	CYFIP2	
	MANBAL	
	MICOS10	
	KRT18	
	APOLD1	
	BPIFA2C	

	SMTNL2	
	BNIP3	
	SLAIN1	
	SGMS2	
	LOC783134	
	PHGDH	
	ELF3	
	CCDC28A	
	TAGLN	
	KCTD1	
	MS4A1	
	CDC42EP3	
	TNFAIP3	
	VPS52	
	PTGER4	
	ARHGEF39	
	MAFG	
	MFHAS1	
	SYT11	
	WDR44	
	MYC	

	LOC617079	
	PMP22	
	OOEP	
	NINJ1	
	CKB	
	EZH2	
	CRACD	
	HHIPL2	
	CEACAM1	
	ENPP5	
	ACADSB	
	TRNT1	
	POU2AF1	
	CLEC3B	
	UCHL1	
	CTSB	
	NLRC5	
	CHST15	
	APOA1	
	ZFYVE1	
	TSEN15	

	SFN	
	RNF157	
	LMNA	
	CCDC82	
	DSN1	
	SNTB1	
	FAM111B	
	YWHAZ	
	ZDHHC23	
	ENO1	
	TMEM45A	
	ACOT9	
	PPP1R3B	
	PCTP	
	NUP214	
	RNF2	
	PGK1	
	NTS	
	VSTM1	
	EFNA1	
	TUBA1A	

	KCNJ2	
	MPEG1	
	CCL1	
	YWHAG	
	LOC101907813	
	STX17	
	NAGPA	
	ASB8	
	COX10	
	TNFRSF12A	
	CHKB	
	BBC3	
	SULF2	
	KPNA3	
	CA8	
	HMCES	
	ABCG2	
	STAT1	
	RAB8B	
	LOC100297152	
	SPINT1	

	GDAP1	
	HK1	
	ANKRD11	
	AMBRA1	
	LIMCH1	
	LRG1	
	DDIT4	
	MBOAT1	
	GAS7	
	AKAP8L	
	PER1	
	PEMT	
	MYNN	
	KLF13	
	RNASE4	
	ACOT7	
	KCNF1	
	FLRT2	
	MANF	
	AGTR1	
	U2SURP	

	HSD17B11	
	PRKCD	
	CELSR1	
	TMEM106C	
	FST	
	AGO2	
	TSTD1	
	FBXO15	
	SLC8B1	
	ASB12	
	NDUFA5	
	STAT2	
	NDRG4	
	SLC26A4	
	LOC100847946	
	HACD4	
	EXOSC8	
	GSPT2	
	IFI27	
	SEPTIN11	
	REXO5	

	IRX3	
	TM7SF2	
	GLIPR2	
	TP53INP1	
	GNAI1	
	SPHK1	
	MOB1B	
	SLC6A17	
	FAM3A	
	EXOSC3	
	MAX	
	LRIF1	
	MANSC1	
	KIF2A	
	LMAN1L	
	ZNF770	
	LOC100295797	
	ITGAV	
	RHBDL2	
	DCTN5	
	HOXA13	

	AQP1	
	ELOVL5	
	GSTM3	
	PCMTD2	
	GNPDA2	
	NKG7	
	CXCL16	
	TMEM120A	
	CIST1	
	ETV1	
	LNP1	
	TRIM6	
	PLEKHO1	
	FYB1	
	TKTL2	
	CMTR1	
	TAP2	
	OBSCN	
	ALPI	
	ANKRD1	
	CCND2	

	HNRNPDL	
	SLC38A11	
	STRA6	
	SPG21	
	EXOSC4	
	NARS2	
	PPM1M	
	PNKD	
	CROT	
	IFIT5	
	LSR	
	ZNF668	
	ETF1	
	TPI1	
	SERPING1	
	PGA5	
	PMVK	
	CLDN7	
	SOBP	
	TRIM37	
	TBXAS1	

	FUBP3	
	LRRC8C	
	TSPAN17	
	PPP2R1B	
	MFGE8	
	HES1	
	BOLA-NC1	
	TOR4A	
	WASHC2A	
	SQSTM1	
	ZAP70	
	CARNMT1	
	SLC66A3	
	SKAP2	
	WIPI2	
	LRAT	
	CXCL3	
	IP6K2	
	ARL8B	
	THBD	
	CSNK1G1	

	LOC327709	
	TCF19	
	WDR77	
	POT1	
	UTP18	
	ACSS2	
	SEC24D	
	CREB3L1	
	CLEC2D	
	DAB1	
	FBXL4	
	HOPX	
	DCK	
	CCDC159	
	CAV1	
	INIP	
	DDX39B	
	RFK	
	RMI1	
	ZBTB1	
	SLC13A5	

	RPS3	
	CTSD	
	M1AP	
	CHIC2	
	ZNF462	
	LOC525599	
	HADHB	
	LOC100138004	
	ARID5B	
	POC1B	
	SELP	
	WASHC1	
	ZNF385A	
	SESTD1	
	SLC46A2	
	SYNJ1	
	RHOB	
	NXPE3	
	DENND10	
	RBM34	
	YWHAB	

	CTPS1	
	JCHAIN	
	SH3BP4	
	EIF2B4	
	CCT4	
	SNX4	
	HPRT1	
	MTFR1	
	WBP2	
	RORC	
	PER2	
	SYTL2	
	SOCS1	
	NCKAP5	
	LOC533307	
	TKDP1	
	ZFP36L2	
	ABHD4	
	SEC24B	
	LTBP2	
	PURB	

	CLECL1	
	TAF1D	
	SPTSSA	
	LOC511936	
	GDAP2	
	CD7	
	CTSO	
	SPON2	
	PIK3R5	
	RENBP	
	ABTB1	
	CNP	
	FBLN1	
	VASP	
	CALML4	
	ARRB1	
	MRTFB	
	GAPDH	
	MZB1	
	RHOQ	
	SARDH	

	NCAPG2	
	SMARCA5	
	GATM	
	ZC3H14	
	DUSP10	
	UBXN8	
	FNBP1	
	GSDMB	
	KIR2DS1	
	FOLR2	
	ZMAT3	
	ECSCR	
	SBNO2	
	MAP3K6	
	YARS1	
	SOCS7	
	FANCM	
	CLDN5	
	LFNG	
	TPD52L1	
	CD37	

	DRAM1	
	DMGDH	
	SELENOF	
	NIBAN1	
	RACGAP1	
	RNF19B	
	LYN	
	MATK	
	PKN1	
	FAT1	
	MACROD1	
	PRM2	
	HNRNPL	
	ASNSD1	
	UPF2	
	TRIM38	
	LEPROTL1	
	LOC530077	
	VIT	
	FAS	
	FMO5	

	TSHZ2	
	LOC100848538	
	BTG2	
	MACROH2A2	
	TYRP1	
	TFE3	
	GRAMD2B	
	PALB2	
	CD300LG	
	PRMT3	
	SLC1A4	
	FAM3C	
	RASAL2	
	TGFB1	
	TMEM41A	
	WDR19	
	MAP1LC3A	
	CCNB2	
	TPBG	
	CCN5	
	RBM12	

	PEG3	
	USP46	
	HCFC1R1	
	LPCAT4	
	DOP1B	
	SNRNP48	
	LOC407198	
	PRL	
	C7	
	TP53I11	
	C1R	
	MIPOL1	
	DHRS1	
	NFKB1	
	TMEM167A	
	OSBPL1A	
	DBNDD2	
	POLD4	
	DHRS11	
	RAP2B	
	MRPS35	

	RELN	
	C1GALT1	
	USP14	
	MRPL40	
	ENTPD6	
	KCTD9	
	DHCR7	
	SNX8	
	RAB26	
	RNPS1	
	DBF4	
	FBXO22	
	F2RL2	
	MGST1	
	CACNB3	
	SLC27A6	
	FGFR4	
	SLCO4C1	
	LAP3	
	MBP	
	CCL8	

	TRANK1	
	ZNF704	
	CBX5	
	PELI2	
	RINT1	
	VAV1	
	LITAF	
	DARS1	
	LOC101904012	
	ACTN1	
	CLCA3	
	ELP6	
	RSPRY1	
	IKBKE	
	LOC540013	
	TFIP11	
	IKBKG	
	OTULINL	
	ZFAND3	
	WDR59	
	IL6ST	

	SLC25A19	
	BZW2	
	TPP2	
	BPNT2	
	SFXN4	
	DGAT2	
	DUSP12	
	DZANK1	
	ARHGEF6	
	ALG6	
	FMO3	
	INTS6L	
	ROR2	
	TRIP13	
	PRND	
	TRIQK	
	PPP1R26	
	LATS2	
	DEAF1	
	NAT10	
	TEX22	

	WDR75	
	PYROXD2	
	DDX39A	
	PSMA4	
	HNRNPA1	
	WASL	
	EHD1	
	PDE8A	
	SAV1	
	CYP1A2	
	MTFMT	
	IGHE	
	NCR1	
	HK3	
	GGTA1	
	CORO1B	
	FAH	
	MGAT5	
	SIRPA	
	CMBL	
	CASKIN2	

	LOC515736	
	IGFBP3	
	C4A	
	LOC100848419	
	FGD2	
	DLX4	
	YY1	
	C1H3orf38	
	ATG14	
	RABEPK	
	LGALS9	
	PAG6	
	ADPRS	
	NDC1	
	PARM1	
	PKP3	
	SMU1	
	TESPA1	
	ZNHIT3	
	NXT1	
	GOLGA4	

	KLC1	
	TDP2	
	TOP1	
	ARHGEF2	
	PFKP	
	MYOG	
	ABCC6	
	KRT15	
	STK10	
	BCL7A	
	TXNIP	
	KPNA1	
	SLC44A1	
	PGS1	
	RND3	
	ECHDC1	
	RFX5	
	NAGA	
	LOC101906240	
	SESN1	
	MEGF6	

	FAM200C	
	MARK3	
	ADAMTS1	
	ACP2	
	CD40	
	CHMP1B	
	PTGIS	
	BOLA-DQB	
	CRTAC1	
	ZNF667	
	CLEC4E	
	CNNM3	
	MAPK1	
	TIGD2	
	BTBD8	
	EYA3	
	EEF1AKMT2	
	GNAI2	
	HSD17B14	
	PEX12	
	ATG4C	

	YPEL2	
	LYSET	
	CD83	
	NCEH1	
	FTH1	
	ZNF275	
	SPX	
	SPSB1	
	LOC104970105	
	TTC27	
	PTBP1	
	RAC1	
	PRKAA1	
	LOC100851369	
	PIBF1	
	RNF114	
	TSPAN1	
	RILPL1	
	TLE6	
	RNF43	
	ATOSA	

	C7H5orf63	
	PTGES	
	MPZ	
	HSH2D	
	CEP19	
	FZD1	
	DEDD2	
	ANTXR2	
	MED30	
	BOLA-DQA1	
	HTR4	
	RET	
	GTF2H4	
	NUP160	
	CD200	
	PNMA1	
	HEBP2	
	ADGRL4	
	XRCC3	
	MEOX2	
	GPN3	

	KCTD5	
	FCGR2A	
	CFAP70	
	GRHPR	
	CCT3	
	VPS26A	
	UBE2R2	
	RAB11FIP2	
	USP13	
	CHST14	
	GPR34	
	CASP2	
	ANXA6	
	RABEP2	
	SLITRK2	
	DNAH11	
	LEPROT	
	LOC614260	
	C1QC	
	WIPI1	
	TRAK2	

	SMIM1	
	MRC1	
	LMNB2	
	PTDSS1	
	SSC5D	
	BACH2	
	LOC782348	
	ZCCHC14	
	EPG5	
	PDCD4	
	EIF3D	
	OGFOD1	
	TMIGD2	
	CHORDC1	
	AMY2B	
	TERF1	
	EIF4E3	
	NENF	
	GPD1L	
	CMTM7	
	PRNP	

	ADRB2	
	ELP1	
	LY9	
	LARGE1	
	KDM5A	
	CHFR	
	LOC528802	
	IL20RB	
	SGSH	
	CEP76	
	ATRAID	
	CD209	
	MSN	
	ATP8A1	
	TEX264	
	HAUS5	
	CATHL1	
	EML6	
	SRL	
	UBE2E2	
	ABCD1	

	GAP43	
	SRP9	
	RBMX2	
	CIDEC	
	NCKAP1L	
	DRC3	
	TLR4	
	ACADM	
	CRY1	
	ARSK	
	HNF1B	
	ACOX3	
	AMELX	
	GPC4	
	LPCAT1	
	LOC100335202	
	PTGES3	
	CRYM	
	SLC16A13	
	PPM1D	
	SPC24	

	SERF2	
	MED17	
	CPNE6	
	TENT5A	
	KLK4	
	CCIN	
	LARS2	
	PIK3CD	
	ACOT2	
	BCAR3	
	CHCHD10	
	CABCOCO1	
	LIAS	
	MTDH	
	ANKRD6	
	LRP11	
	FRG1	
	PRC1	
	TMEM171	
	ST14	
	PLAUR	

	CAST	
	TIMP3	
	LAIR1	
	TIE1	
	MOV10	
	CATSPERG	
	SBF1	
	SLC50A1	
	LOC101904013	
	WDTC1	
	CST3	
	CD276	
	BLMH	
	HCRTR1	
	EIF3B	
	SORL1	
	RRP12	
	TAGLN2	
	SORCS2	
	PCOLCE2	
	FAM83G	

	EAF1	
	SMS	
	ABCB10	
	GRWD1	
	ZNF75D	
	DONSON	
	C15H11orf58	
	GRIPAP1	
	SEC23A	
	DENND2D	
	PRIMPOL	
	TSTD3	
	LDLRAD4	
	SFTPD	
	ARMCX5	
	COL17A1	
	UBE2F	
	TCIRG1	
	SEC11C	
	CD101	
	CSF1R	

	MAP3K7CL	
	ENPP1	
	SIAE	
	CLIP2	
	ARRDC2	
	SNX6	
	HSPB8	
	MCM7	
	NT5C3A	
	GATA6	
	SOD1	
	TPM2	
	TRPC6	
	C2	
	FKRP	
	PTPN6	
	RPS11	
	ZFAND5	
	EIF2S2	
	PLAGL1	
	UBE2L6	

	CCDC70	
	HTR1A	
	LOC522845	
	LRRC46	
	FOXL2	
	CHAF1B	
	SELENOM	
	SRRT	
	UTP4	
	MAPK6	
	CDCA3	
	PLAA	
	CYB5R4	
	SPARCL1	
	UBE3B	
	TUBGCP6	
	SERPINF2	
	SLF1	
	RALGPS1	
	VIPAS39	
	FXYD1	

	RUSC2	
	CCNJL	
	METTL14	
	SYNE4	
	COL10A1	
	CAPG	
	DHX32	
	SLC5A1	
	FOLR3	
	ATL3	
	PDK3	
	RDH12	
	SHMT2	
	MYOF	
	SREBF1	
	ETHE1	
	FIBIN	
	ZNF236	
	FGFRL1	
	AMMECR1L	
	HINT3	

	DPH5	
	H2AX	
	SGO2	
	ACLY	
	NRP1	
	MYBPH	
	STYX	
	ARMC7	
	BCL6B	
	VWA8	
	PRR5L	
	SOD3	
	LOC100848339	
	ATP2C2	
	KLF10	
	GRB7	
	HERC1	
	C26H10orf88	
	SLAMF7	
	VPS13D	
	MYO5B	

	RBM14	
	ZRSR2	
	KIZ	
	ANO6	
	SLC35D2	
	AMOT	
	BHLHE41	
	LOC516599	
	CDKN2AIP	
	PLPP6	
	RIOK1	
	TRIT1	
	CAMTA2	
	CD93	
	PSME3IP1	
	FCGR1A	
	MYRIP	
	ABL1	
	HRG	
	ARPC4	
	C6H4orf54	

	WWC1	
	RASGRP2	
	USP2	
	AADAT	
	BUD13	
	CA4	
	YIPF1	
	NR3C1	
	DR1	
	C1QA	
	ZNF839	
	B3GNT4	
	GPR162	
	ANGPTL8	
	ZNF181	
	GDF7	
	GNAI3	
	IRF3	
	AGPAT1	
	CDCA7	
	MPL	

	TMEM220	
	YJU2B	
	TMEM250	
	IL15	
	PLSCR2	
	VARS1	
	NME4	
	PAPOLG	
	AFF1	
	RFC5	
	GALNT13	
	ATP6AP1	
	JSP.1	
	WWP2	
	PPP3R1	
	EDRF1	
	JUP	
	CLIC2	
	DPP3	
	ELMOD3	
	ELF5	

	C15H11orf52	
	GPT	
	CDK8	
	ABCC5	
	SBDS	
	TENT5B	
	CAPSL	
	PITPNM1	
	PPME1	
	PARVB	
	TRPC4AP	
	GALNT1	
	TNFSF13	
	CD163L1	
	FAM161A	
	IGSF3	
	PDZD2	
	SCD	
	ANO1	
	GCNA	
	DNPEP	

	CNTFR	
	C1S	
	SYNPO	
	INTS7	
	ARMCX3	
	PBDC1	
	KAT2B	
	OR10G1C	
	HFE	
	KANSL2	
	PPID	
	TNFRSF21	
	NRBF2	
	LOC617281	
	RGCC	
	TACC3	
	GATA3	
	BCAS1	
	LOC511531	
	SLC35A5	
	WDR73	

	STEAP2	
	SMIM3	
	IGL@	
	SDAD1	
	RIOK2	
	USP19	
	UBA7	
	TEF	
	ATXN3	
	PELI1	
	LOC101906836	
	VTA1	
	CEP44	
	MCUR1	
	RIGI	
	EML3	
	PABIR2	
	LRRC75A	
	CD1D	
	LHFPL3	
	BCL6	

	CCDC6	
	KAT14	
	GPI	
	PDZK1IP1	
	SLC25A38	
	AZIN2	
	SLC25A29	
	RBM12B	
	ETFB	
	RBBP8NL	
	OCIAD2	
	DNASE1L1	
	HTT	
	TEX10	
	B4GAT1	
	CCM2	
	TRIM54	
	CHGA	
	ATP10A	
	FNDC7	
	FAM20A	

	RTCB	
	PHF24	
	SLC5A6	
	BRINP1	
	SMCO4	
	ENTREP1	
	RCN3	
	CBR1	
	UBA6	
	ADHFE1	
	ELOVL7	
	CPEB4	
	SMAGP	
	SERPINF1	
	CUL2	
	USP15	
	CLCN7	
	ADAMTSL3	
	CACNA1B	
	FGFR1OP2	
	VPS33A	

	PARP4	
	TXNRD1	
	NSD3	
	TGFB3	
	PLCL2	
	SOCS4	
	NFIB	
	TOB1	
	EXO5	
	RAPGEF2	
	COPS2	
	KLF11	
	TRIM28	
	GMNN	
	KCNE4	
	VPS51	
	ENPP2	
	LOC512672	
	SGPL1	
	FNDC11	
	BOK	

	GPR68	
	CCT2	
	HIP1	
	LCAT	
	EGFL7	
	KCTD18	
	DAPL1	
	PSAP	
	SLC39A1	
	KIAA1191	
	MAPKAPK3	
	BPI	
	RADX	
	SLCO2A1	
	SLC38A3	
	FBP1	
	HOXD10	
	FAM83F	
	TUSC3	
	CMTR2	
	SUSD6	

	TRMT61A	
	VPS50	
	ZBTB14	
	ERBB2	
	CHRM4	
	SLC66A2	
	GLI3	
	LTA	
	MRPL44	
	TPM4	
	USP25	
	KLHL29	
	ILF3	
	BMP1	
	SCNN1B	
	MKLN1	
	ADGRB2	
	GREB1	
	BDH2	
	UAP1L1	
	PPARGC1A	

	DHX58	
	CPT1A	
	OS9	
	GSTO1	
	SH3PXD2B	
	ZC3H8	
	LOC280930	
	DNMT3B	
	UXS1	
	HNRNPLL	
	NRK	
	C16H1orf159	
	RETREG3	
	TNFAIP1	
	HHAT	
	GPR61	
	CADM1	
	C1QB	
	CPNE8	
	ZC2HC1A	
	PID1	

	ARAP1	
	DNMT1	
	RCN1	
	EFCC1	
	LDOC1	
	PPP1R18	
	TTI2	
	ACOX1	
	BCAN	
	TSPAN32	
	WDFY1	
	DDAH1	
	CLHC1	
	TOE1	
	HCFC1	
	MTHFR	
	SLC22A8	
	ARL6	
	MYD88	
	SMIM10L3	
	DDX41	

	NTMT1	
	HAX1	
	SHROOM3	
	MYH11	
	OGT	
	LAPTM4A	
	CKMT1A	
	RHOJ	
	PTPN12	
	SPMIP6	
	NSG1	
	LGALSL	
	ZNF518A	
	TMT1A	
	FAM181A	
	PIK3C3	
	TCERG1	
	IL27RA	
	PLCG2	
	CDS2	
	NCOA7	

	MSMB	
	STIL	
	RABEP1	
	TMEM187	
	WIPF3	
	TSR1	
	SCAF8	
	NHSL1	
	BCL2L15	
	BAG5	
	CERS5	
	EFTUD2	
	BCL3	
	TARS1	
	ACTR3	
	XPNPEP2	
	PKIB	
	TFDP1	
	THOC5	
	CAPN10	
	PPP1CA	

	PNLIPRP2	
	PSMD13	
	DAG1	
	PDGFC	
	ORMDL3	
	PQBP1	
	NRN1	
	CHAD	
	CCDC112	
	TPM1	
	MBNL3	
	POLR3F	
	TPD52	
	PIK3CA	
	PAFAH2	
	GSTT1	
	CHURC1	
	GSG1L	
	NPPA	
	ABLIM3	
	ADCY7	

	SYNGR1	
	NOC2L	
	SMAP2	
	SFXN3	
	RORA	
	HSPBAP1	
	ASCL2	
	QPCTL	
	BCAP29	
	CRHR2	
	ACTA2	
	RALGDS	
	ACSM5	
	DNAJC9	
	FLOT1	
	PPP1R32	
	RPH3A	
	VPS36	
	RELB	
	CD2BP2	
	STK17A	

	PTPN2	
	KIAA0232	
	TJP2	
	IRF7	
	KRT17	
	DKK4	
	CD164	
	NSMCE4A	
	IMPDH1	
	FSTL1	
	TOX	
	GM2A	
	COL4A4	
	MAP2K6	
	CDK1	
	AKR7A2	
	LOC101908745	
	SFTA2	
	BOLA-DMA	
	HDAC4	
	NUP133	

	RYK	
	PICALM	
	SLC38A1	
	TMEM86A	
	PPFIA3	
	PEX11A	
	GOT2	
	ELAVL3	
	BHLHE40	
	KRT78	
	LOC504295	
	MFAP5	
	GPRC5C	
	TMEM199	
	SDR16C5	
	CAP1	
	KIAA2013	
	GTF2H1	
	CORO2A	
	P4HA2	
	JAKMIP1	

	USP32	
	TCHP	
	PRDM1	
	MALL	
	IGSF5	
	CREG1	
	DPH2	
	UBE2L3	
	LOC101903253	
	ATP1B3	
	MYCN	
	ACVR2B	
	EPHX1	
	TBXA2R	
	GCLM	
	CLDN12	
	PSMG3	
	YBEY	
	PTCD2	
	ULK1	
	RPS4Y1	

	SIX4	
	DUSP3	
	HSPA4	
	PPIL1	
	ADGRG5	
	RNF13	
	ZC3H6	
	PSMA1	
	DSG2	
	FKBP15	
	PPARA	
	SAMM50	
	IMPDH2	
	EHF	
	ZBTB47	
	MBD2	
	PTPMT1	
	NMI	
	TMEM144	
	STAT6	
	IL21	

	TRIM5	
	ATP6V1C1	
	PCBP2	
	TTPA	
	BOD1L1	
	CLDN16	
	ZNF341	
	MLEC	
	LOC100297097	
	TSN	
	PTPRG	
	GHDC	
	CCDC90B	
	ACTR10	
	SMPD4	
	RSL1D1	
	AFAP1L1	
	FBXL5	
	ANKRD12	
	ALG3	
	TMEM178A	

	FHOD1	
	MCRS1	
	PLEKHG1	
	CHAMP1	
	TAF12	
	RUNDC1	
	TP53	
	TGFB1I1	
	GMFB	
	TMEM140	
	ABT1	
	ZCCHC8	
	LDAF1	
	PBX3	
	SLC25A37	
	KCNE1	
	TFRC	
	ATP6V1H	
	IL4R	
	PPP1CC	
	CALHM6	

	IST1	
	SLBP	
	TMEM203	
	MIR29B-2	
	PDLIM7	
	LEMD2	
	ME1	
	PYGL	
	CRABP2	
	MAPK13	
	BREH1	
	CPLX2	
	RPL32	
	LY6G6E	
	FASTKD2	
	MICAL1	
	ARF3	
	GTF2F2	
	DPY19L1	
	BRD3OS	
	COPZ2	

	SOX18	
	ARHGEF10	
	NRXN1	
	TIAM1	
	SULT1A1	
	NEDD4L	
	SCLY	
	AQP7	
	CHMP1A	
	TTF2	
	IFRD1	
	FOXK2	
	SERPINH1	
	ATP2A2	
	LTBR	
	SDCBP	
	SERBP1	
	AGFG1	
	COL14A1	
	CYB5B	
	MYLK2	

	LOC100848598	
	DYNC2H1	
	PROCR	
	PNRC2	
	DCT	
	TEKT1	
	RARS1	
	DNAJC10	
	SDC1	
	PSMD5	
	KLF2	
	UTP15	
	PF4	
	SORBS3	
	FAM114A2	
	FBXW2	
	PDCD11	
	EPAS1	
	CD320	
	NIPAL2	
	CBFA2T2	

	VPS37C	
	HDDC2	
	TTI1	
	TANK	
	GTF2H3	
	PIGV	
	ALOX12B	
	GRIK5	
	GUCY2F	
	FRMD3	
	ARSB	
	IP6K3	
	PUM1	
	MDC1	
	PAX8	
	EGLN3	
	DDX20	
	CRIM1	
	LARP7	
	AHNAK	
	SNAPC1	

	ANP32E	
	RHO	
	NR1H3	
	TRMT10B	
	SLC12A7	
	PTGFRN	
	CSPG4	
	FLCN	
	LOC100337293	
	UBE2O	
	SOCS2	
	ABI1	
	RCAN1	
	PARD6B	
	CA12	
	SLC25A40	
	SIK3	
	B4GALT4	
	CNOT8	
	PECAM1	
	ADGRD1	

	PLN	
	FADS6	
	TMEM218	
	LYAR	
	CPNE2	
	MRPS18A	
	GHRH	
	SRGAP2	
	RAB32	
	GOLT1A	
	PRKCA	
	C23H6orf226	
	ADGRL1	
	CD8A	
	RFTN2	
	SS18	
	ARFRP1	
	PRKAR2B	
	HRAS	
	FLNC	
	KAZN	

	PARD3B	
	RPAP1	
	METTL25	
	DTX2	
	SMOC2	
	TMCO3	
	TDG	
	UBE2J1	
	WDR54	
	CDH26	
	USPL1	
	RBM45	
	SCARB1	
	PREP	
	MALT1	
	PANK1	
	NPEPL1	
	MPPED2	
	SH3D19	
	POLG2	
	RPIA	

	GIMAP8	
	KCNH3	
	STC1	
	ATAD5	
	CUL4A	
	SLC35E3	
	NOS2	
	LIPE	
	NUP37	
	TBC1D16	
	C17H12orf76	
	TBC1D17	
	ATF6B	
	FKBP7	
	TRIM8	
	NT5DC1	
	IL17RB	
	TIMM9	
	PGPEP1	
	MAMDC2	
	CHRM1	

	SKA1	
	TUT7	
	NKRF	
	URI1	
	KAT2A	
	TMEM132A	
	CSNK1D	
	NKAPD1	
	TTC7A	
	NUDT15	
	GIN1	
	IKZF1	
	RAD54B	
	CERT1	
	LRRC34	
	TNS2	
	EEF1AKMT1	
	CPSF6	
	CSNK1G2	
	IFFO1	
	FGB	

	LOC515578	
	SNX2	
	HTR5A	
	CSAD	
	TLR7	
	CD81	
	SLC25A51	
	CLXN	
	OPCML	
	GP2	
	COX8B	
	CTTN	
	IFFO2	
	NXPH3	
	SLC37A1	
	FBXL17	
	ZXDC	
	LYNX1	
	RAB5C	
	SLC43A1	
	SLC5A11	

	CPA1	
	TKT	
	FARS2	
	SCPEP1	
	PUS7L	
	LAMC2	
	TLR9	
	GMCL1	
	SERINC5	
	KIFAP3	
	CNST	
	CMTM4	
	RASSF5	
	TLNRD1	
	UBE3C	
	PSMD12	
	EPB41L4A	
	MED22	
	PATZ1	
	PDCD7	
	MED28	

	TCEA1	
	HNRNPF	
	NIPAL3	
	ADORA1	
	LOC523373	
	AKNA	
	SLC45A1	
	VEZT	
	ITPR1	
	CRLS1	
	XPO1	
	ATAD2B	
	LOC512150	
	RNF4	
	DDX55	
	KMO	
	LOC101904413	
	MRPL12	
	NUS1	
	TMEM232	
	RPRD1B	

	ETS1	
	STX4	
	GPA33	
	OPN1SW	
	PNPLA7	
	DGAT1	
	SORT1	
	SLC13A2	
	NEO1	
	ZSWIM7	
	TMEFF1	
	GGT1	
	PPP2R1A	
	TLCD3A	
	ARHGAP9	
	BTBD1	
	BTC	
	UBAP1	
	PDZD8	
	FOXP3	
	TMEM200B	

	MGC137014	
	DYRK2	
	FAM219B	
	F2R	
	CLN8	
	PCDHA13	
	SIRT3	
	CYLC1	
	SLCO3A1	
	CPSF1	
	IHH	
	DAPK1	
	CBX2	
	GID4	
	MEGF9	
	ZSWIM6	
	TRGC4	
	TMEM38B	
	ZBP1	
	ASGR1	
	UNC45B	

	TARS2	
	PNPLA2	
	PINX1	
	TOM1L1	
	SKIL	
	FAM81A	
	ZNF503	
	FETUB	
	PWP1	
	TMEM168	
	CXCL17	
	FBXO5	
	CEL	
	ADSL	
	APOOL	
	ZNF239	
	IFNAC	
	VTI1A	
	TRAF1	
	RFLNA	
	NUP107	

	NFKBID	
	COA1	
	DCAKD	
	CYLD	
	FIGNL1	
	CD248	
	PGAM1	
	CEP95	
	DUSP16	
	ADAM8	
	PDLIM3	
	SLC4A8	
	EEF2	
	TFF2	
	ARMCX1	
	RBMS1	
	SPATA20	
	NTRK2	
	DMAC2L	
	ZNF567	
	PDE7A	

	NAALADL1	
	MYO18A	
	PLXNB2	
	ISCA1	
	PRKDC	
	VGLL1	
	ZNF582	
	RBBP5	
	MYH10	
	ORC3	
	DTX4	
	NAAA	
	IDI1	
	COIL	
	TLR5	
	TRNAU1AP	
	GALNT17	
	IRF5	
	PTRH2	
	TUBE1	
	CHTF8	

	CEP63	
	UPF3B	
	DDX52	
	GALNT14	
	LOC100847345	
	KRT5	
	REG3G	
	LOC100335819	
	SCAF11	
	TBC1D15	
	HERC4	
	CYTH4	
	LAMP3	
	GPX7	
	KIAA1549	
	LSM6	
	SART3	
	ATN1	
	CTR9	
	NAA40	
	ARL16	

	METTL27	
	RASAL1	
	CALR	
	ROGDI	
	RIOK3	
	CTTNBP2	
	NCOR2	
	BPIFA1	
	FGD3	
	AMBP	
	LOC100847947	
	MYBL2	
	EDN2	
	IER5L	
	CNOT6	
	ACADVL	
	CYB5D2	
	DHRS4	
	PDSS2	
	DSC2	
	CAVIN2	

	DNASE2	
	CORO7	
	GTF3C1	
	WDR45B	
	CHEK1	
	LOC539876	
	RPGRIP1	
	LOC100297608	
	TNS4	
	PRICKLE1	
	LOC508285	
	FMNL3	
	LRRC25	
	ANGPT1	
	COL2A1	
	MCOLN1	
	TBX19	
	ASPA	
	EIF4A1	
	GABRP	
	ALG5	

	SLC5A2	
	BICRAL	
	YWHAH	
	LOC618071	
	PRIM2	
	CRYBA4	
	NADK	
	ZNF330	
	MYO9B	
	SLC20A2	
	REEP1	
	COPZ1	
	POLR1F	
	SEMA4F	
	SLC22A5	
	ELAPOR1	
	BRD1	
	UBE2Z	
	ZFHX4	
	MICU1	
	ABCA5	

	CNRIP1	
	ITGAL	
	ASCC1	
	LOC508646	
	TFF1	
	FASTK	
	HAT1	
	PDPR	
	ADAMTS8	
	IL2RA	
	ATF7IP	
	KIF5C	
	COL6A1	
	PITPNA	
	MICAL3	
	HEATR6	
	CCND1	
	ZMIZ2	
	SIRT7	
	LOC407165	
	BANP	

	THOP1	
	MTIF2	
	FIGNL2	
	GPR37	
	DFFB	
	SYNRG	
	PLIN5	
	SMARCD1	
	TM4SF1	
	HAND1	
	ITPRID2	
	NUP43	
	ATF2	
	SPAG1	
	CSNK1B	
	QTRT1	
	SART1	
	LSP1	
	PRADC1	
	LOC100295883	
	ACSM2B	

	ABCA3	
	IL13	
	CAPN2	
	WWP1	
	TAF6L	
	BFAR	
	GUK1	
	ADGRG7	
	MRC2	
	FXYD3	
	GYS1	
	TUBG1	
	CYP39A1	
	CYCS	
	GNA15	
	STMP1	
	TBCE	
	FGF14	
	ENOX1	
	ACVR1B	
	EPB42	

	ASCL1	
	FHDC1	
	CAMKK1	
	AHDC1	
	LOC786530	
	TMEM62	
	PROX1	
	KNL1	
	HSPA12A	
	PADI2	
	CPN1	
	AVL9	
	RTL6	
	ATP10B	
	TRIM52	
	FEM1A	
	C16H1orf116	
	CXCR6	
	TMEM185B	
	CYP1A1	
	KIFBP	

	ZNF608	
	QSOX2	
	CCDC117	
	ENOSF1	
	TBCEL	
	ZNF207	
	RNF19A	
	LOC514189	
	SLC39A6	
	PLSCR3	
	BOP1	
	OMA1	
	AEBP1	
	PAG10	
	AK3	
	ASB5	
	ADAMTSL2	
	ZDHHC7	
	PALMD	
	VSX1	
	AMBN	

	ARAF	
	LOC509961	
	KIDINS220	
	C6	
	SIDT2	
	ATP11A	
	NELFE	
	PSRC1	
	SNX11	
	DNAJC17	
	SOCS6	
	TRMT6	
	RRAGB	
	EXOC3L1	
	MR1	
	NOXA1	
	MET	
	NUDCD1	
	LAMA5	
	MAST4	
	DNAJB5	

	COQ5	
	SERPINB6	
	CBLC	
	PLRG1	
	SECISBP2	
	MFNG	
	TUBB6	
	LAMP1	
	TMEM42	
	PIGW	
	TMEM131L	
	ZNF706	
	GXYLT1	
	PPL	
	MIR221	
	CPSF3	
	PPP4R1	
	LUZP1	
	PPP1R42	
	RNF149	
	LOC100847661	

	SF3A3	
	NUP50	
	LOC509972	
	PDE6G	
	SOX2	
	WDR72	
	DNAH9	
	SEMA6A	
	TBCD	
	RGS2	
	PNN	
	CYP7B1	
	MMP15	
	ZNF316	
	COTL1	
	TBL1XR1	
	PLVAP	
	ARL4A	
	NOSIP	
	PLIN3	
	TCRG	

	CDC5L	
	IL20RA	
	TXNRD3	
	RSU1	
	RMND5A	
	SHANK2	
	MAPRE2	
	ARGLU1	
	CA11	
	ORC2	
	IRS1	
	LOC101908182	
	RASGEF1A	
	ENPEP	
	ARID3A	
	TLE5	
	ADCY6	
	PPP1R3D	
	FAM43A	
	UTS2R	
	SLC1A5	

	SATB1	
	CMTM8	
	AIDA	
	CNBP	
	PRR15	
	VAMP3	
	ATP6AP2	
	GNG3	
	OTUB1	
	GINS3	
	SLC26A6	
	GPR176	
	SLC43A2	
	CNN3	
	METTL13	
	ADAM1B	
	PIP5K1B	
	PUM3	
	LOC786796	
	MYOZ1	
	NOTCH2	

	REV3L	
	PPM1G	
	RMND1	
	MLF1	
	ALDOA	
	ZNF572	
	EPHX3	
	NEK9	
	RPS6KA5	
	PGGT1B	
	CIC	
	TMEM50A	
	CYP19A1	
	TICAM2	
	UCKL1	
	CD1B	
	SLC36A4	
	ODF1	
	KANSL3	
	PAH	
	CSPP1	

	SPAG4	
	ATP6V0E2	
	DDIT4L	
	SFT2D3	
	DUSP13	
	LRP2	
	CATHL2	
	HCN4	
	SLC9A1	
	MYSM1	
	MRPL15	
	MFSD5	
	IGIP	
	PAICS	
	PLAC9	
	HSPA14	
	MEIS1	
	EPSTI1	
	RAP1GDS1	
	ZNF23	
	TUBD1	

	KAT6B	
	TTC1	
	VPS29	
	DLK1	
	PAM	
	CFAP119	
	ST8SIA1	
	TBL3	
	KEL	
	BEX4	
	AKT1	
	CHCHD7	
	ATP6V0B	
	YIF1B	
	CTNND2	
	TOX2	
	LZTR1	
	MARK2	
	SSC4D	
	KDM1A	
	USP3	

	GKN1	
	HMGA1	
	KCNQ1	
	KIRREL2	
	NRP2	
	SPAG9	
	STMN3	
	MRPL36	
	PGLYRP1	
	MRPL46	
	SEMA5A	
	ASH2L	
	CEP57	
	TCIM	
	CD84	
	VTI1B	
	SULF1	
	RAB31	
	OSBP	
	PSMD2	
	KLHL11	

	ATP4A	
	PLXDC2	
	SLX4IP	
	MCM10	
	AP2M1	
	TBC1D4	
	SSTR5	
	APOBEC3Z2	
	SETX	
	PRPH2	
	TCL1B	
	THOC3	
	RPL7	
	POLR3D	
	GCM1	
	USP7	
	MAN2C1	
	PCSK6	
	PAXIP1	
	NUTF2	
	CCT5	

	AKIRIN2	
	SLC8A1	
	SLITRK6	
	STX7	
	EMC1	
	CAPRIN1	
	HOXC11	
	PKP4	
	DCPS	
	DANCR	
	TNRC6C	
	PAMR1	
	KITLG	
	LAMA4	
	ATP13A3	
	DCBLD1	
	VAMP7	
	USP8	
	CDR2	
	PITPNC1	
	PDHX	

	PRMT2	
	RELL2	
	NPY2R	
	GLYAT	
	GDPD1	
	ACTA1	
	ATP1B1	
	CLUAP1	
	ELFN2	
	BOLA-DMB	
	TM7SF3	
	CCNT2	
	NCK1	
	GGH	
	NICN1	
	EBP	
	RHOU	
	SHQ1	
	FLT4	
	PIK3C2G	
	RNF216	

	NPNT	
	GRK1	
	PEPD	
	PLK5	
	ADAMTS2	
	LHB	
	RIPK2	
	BAK1	
	TCFL5	
	ALAD	
	NMT1	
	LOC511175	
	MPHOSPH6	
	ST7L	
	GYPC	
	SWAP70	
	DNASE1L3	
	ZNF470	
	CALM1	
	CDK5RAP1	
	ABHD8	

	HABP2	
	ACTR6	
	CABP2	
	ALKBH8	
	LCOR	
	GATA5	
	HDAC6	
	ARHGAP12	
	DPYSL2	
	LOC518526	
	TDRD7	
	RCSD1	
	RBM42	
	BUD23	
	PRRT4	
	GLE1	
	TRIP10	
	ALDH9A1	
	CRABP1	
	ZNF175	
	PARS2	

	CATHL6	
	ZNF414	
	FAM149B1	
	CRYBB3	
	NPDC1	
	DUSP8	
	TAF11	
	TK2	
	CSNK1A1	
	GUSB	
	MC4R	
	SMAD7	
	PANK2	
	TK1	
	ATP13A5	
	ADSS1	
	TMEM100	
	ZNF410	
	CCDC162P	
	ANKRD23	
	PRKCH	

	GC	
	LOC511240	
	KIR2DL5A	
	ANO2	
	TUFT1	
	HNF4A	
	RNF31	
	BLOC1S5	
	PVR	
	IER2	
	PHF23	
	MEI1	
	CLDN23	
	RIT1	
	RPS6KB2	
	SEPHS1	
	COL4A1	
	TRDMT1	
	LOC505658	
	SURF6	
	ACSL3	

	MAK16	
	MLLT3	
	ATP1A2	
	ELMO3	
	RAB17	
	DDHD2	
	NUP93	
	DCTN1	
	RSBN1	
	DCTN4	
	NABP2	
	BABAM2	
	TSC2	
	OVCH1	
	ARHGAP18	
	AFP	
	JAGN1	
	HS3ST5	
	MINK1	
	NOL10	
	SERPINI2	

	OSBPL7	
	ZIC2	
	LHX3	
	LOC503858	
	UBE2N	
	ACSS1	
	ZMAT5	
	SEMA4B	
	SLC35B1	
	ARID1B	
	HINFP	
	GAMT	
	NRIP3	
	SNAPIN	
	LETM1	
	VIM	
	JRKL	
	RSRC2	
	MDM2	
	CHRM5	
	MAP3K2	

	ABCA4	
	CFL2	
	FNIP2	
	ANKLE2	
	GNMT	
	GARNL3	
	MCM3	
	LOC100335608	
	CARTPT	
	ARRB2	
	CLDN19	
	FILIP1L	
	SNX16	
	TUBA4A	
	SORD	
	MOCS2	
	VSIR	
	ERMARD	
	LOC100848149	
	WDR87	
	TNKS1BP1	

	ODAD1	
	EXOC3L2	
	SPNS1	
	NPBWR2	
	TKDP4	
	PPP1R13B	
	RDM1	
	WDSUB1	
	SMC1A	
	TMEM14A	
	NIPA2	
	ALDH6A1	
	FSHR	
	GPALPP1	
	RESTA	
	CD99	
	CDC42SE2	
	FKBP10	
	FCGBP	
	GJB1	
	CSRP2	

	SCARF2	
	ERCC3	
	ENTR1	
	SLC25A4	
	SELENOO	
	SYK	
	MVB12B	
	SLAIN2	
	CDK19	
	ANXA11	
	TMCC1	
	UBP1	
	CCDC127	
	PIMREG	
	MAP2	
	CD151	
	DYDC2	
	HNRNPUL1	
	ENDOD1	
	TRPS1	
	NHS	

	TOR1B	
	OPRM1	
	SERPINC1	
	FMO4	
	CTNNBL1	
	METTL6	
	NUP85	
	SLC10A3	
	DENND5A	
	HNRNPA0	
	LIMS1	
	SRPK3	
	SLC35G1	
	ALCAM	
	TAPBP	
	C14H8orf33	
	ATP6V0D1	
	MMD	
	LRCH1	
	GULO	
	PYROXD1	

	DRD2	
	TNNC1	
	EIF6	
	MYADML2	
	STAM	
	PSMD7	
	MCU	
	ZDHHC22	
	CIITA	
	IRAK4	
	PWWP2A	
	FXYD7	
	SGCA	
	NRG2	
	PUSL1	
	NT5DC2	
	FOXN1	
	STAP2	
	SAP30L	
	GLCE	
	WIPF2	

	NAPEPLD	
	PRPS1	
	SYT3	
	NOS3	
	DIXDC1	
	RAB34	
	CHEK2	
	LOC407145	
	RASA3	
	PEAK1	
	AK4	
	DCAF15	
	RABL2B	
	DBH	
	MB	
	IDE	
	PPP1R27	
	MTRR	
	HBM	
	ABO	
	HSPG2	

	NNT	
	IARS1	
	KLHL42	
	C1QBP	
	RAPH1	
	IQCD	
	LDB3	
	SMPD2	
	SF3B2	
	CASKIN1	
	RMND5B	
	LYST	
	TIMP2	
	GZMB	
	KDM1B	
	MYO15B	
	LOC100848883	
	WASHC4	
	PAPSS1	
	ATG12	
	GALNT16	

	TBKBP1	
	HPS3	
	JAM3	
	RPS2	
	ACER3	
	DEDD	
	APOBR	
	TRIM21	
	GLUD1	
	SEH1L	
	POLR1E	
	CAMSAP1	
	MGC127133	
	KCNRG	
	ORAI3	
	KLF15	
	NUFIP1	
	ZCCHC12	
	HIPK4	
	KBTBD11	
	RIPOR3	

	SLC2A1	
	CYP4V2	
	ISG20L2	
	CCER2	
	EIF2AK4	
	LOC786987	
	CYBC1	
	HTR2A	
	KIAA0319L	
	EP400	
	PEG10	
	RNF220	
	LOC783020	
	DMTN	
	NPM1	
	PLPBP	
	LOC511425	
	ARPC2	
	HMGN4	
	PROK2	
	CD82	

	JAK3	
	INPP5K	
	GGCT	
	FBH1	
	TTC39A	
	GNL3L	
	IL12RB2	
	TMCO4	
	TFCP2	
	TNS1	
	NDFIP2	
	MARVELD1	
	SGPP1	
	DOK2	
	OSMR	
	GRK6	
	PREX1	
	FANCG	
	GALNT12	
	NSMCE3	
	BRAT1	

	RHPN1	
	FRY	
	MEIS2	
	MYOD1	
	CARS2	
	TMEM216	
	TNNT1	
	AKAP6	
	VPS41	
	GTSF1	
	MTR	
	SSNA1	
	KCTD6	
	IFT122	
	MTRF1L	
	C15H11orf16	
	DLX5	
	MMP17	
	NEDD1	
	ABCB8	
	CLDN6	

	DPAGT1	
	PAG12	
	SERTAD1	
	DBN1	
	ALKBH4	
	FSCN1	
	CABP4	
	CNDP2	
	SPAM1	
	METTL1	
	POLR1H	
	ADH5	
	RRAS2	
	GCNT3	
	HLCS	
	FHL1	
	LOC530031	
	SFTPC	
	MYO15A	
	PXYLP1	
	HSPB1	

	LPIN2	
	TMC4	
	RBM28	
	ESPL1	
	PERP	
	RP2	
	RRN3	
	PAG18	
	ZNF436	
	ANKRD16	
	FOSL2	
	RAB29	
	MST1R	
	ARMC8	
	MTHFS	
	HOXB8	
	LOC518495	
	PRKAA2	
	SPICE1	
	TPCN1	
	LOC784866	

	ARFGAP1	
	CCR7	
	THRB	
	MSL1	
	ATG7	
	AURKAIP1	
	TCAF2	
	BAMBI	
	CRYBA2	
	TMEM129	
	FOLH1	
	KTI12	
	PGAM2	
	BFSP1	
	SNCG	
	BCAR1	
	CLSTN1	
	CAMP	
	SMG7	
	ZFX	
	CD180	

	ALDH1L1	
	TOR3A	
	UBXN1	
	NUDT9	
	WDR13	
	KRTCAP3	
	TM2D3	
	SMARCB1	
	SHH	
	CD6	
	GIGYF2	
	SMC4	
	METAP1	
	ZNF74	
	CCDC174	
	CCNO	
	LOC785873	
	ANKRD34A	
	NALF2	
	MPP1	
	IRF6	

	SYP	
	PRKCG	
	SIGLEC1	
	LRP10	
	LOC100847495	
	HMGCS2	
	LOC783956	
	UGT2A1	
	FAM210B	
	MITF	
	MYO19	
	PLCXD2	
	GOLM1	
	P3H1	
	ABCC8	
	RNF139	
	HDHD2	
	ALDOB	
	CTNNBIP1	
	MIOS	
	LOC100848815	

	UCP2	
	SCML4	
	BRD8	
	FGFR3	
	KCNB2	
	LIF	
	ITGB7	
	BORA	
	NME6	
	LYPLA1	
	ATRN	
	VAC14	
	SHC3	
	CL43	
	ADAMTS6	
	CHST8	
	AKR1B1	
	CD58	
	IKZF3	
	FHAD1	
	TSPAN15	

	OGFRL1	
	RPA1	
	ALDH1L2	
	NTPCR	
	SAMD8	
	ZFP57	
	TNFSF13B	
	PDIA3	
	INTS4	
	HNRNPH2	
	BOLA-DOA	
	PSMF1	
	NACC1	
	NEK1	
	MECOM	
	PAIP2B	
	CUEDC2	
	LY6G6C	
	MC1R	
	SLC40A1	
	GET1	

	GTF2IRD1	
	RBM33	
	CENPN	
	RETREG2	
	CD79A	
	MKRN1	
	PSMD1	
	MED1	
	TSPY	
	TRMO	
	HBP1	
	MTARC2	
	HDAC5	
	SLC7A9	
	SLC4A1	
	DIP2B	
	SLC35F2	
	STRN3	
	SLC6A3	
	CNNM2	
	ARL3	

	TMEM19	
	PNLIP	
	TTC12	
	KIF16B	
	OPN1LW	
	LRWD1	
	CYP17A1	
	ZNF184	
	PITPNB	
	XDH	
	USP48	
	NDUFA4	
	TRAPPC8	
	TRAM1	
	DPP10	
	CADM3	
	RNF144B	
	LOC512863	
	TNP1	
	INTS9	
	FUT8	

	RASSF1	
	ANK1	
	GRN	
	DOCK6	
	SPTY2D1	
	GTF2E2	
	MIGA1	
	TLCD1	
	HIF1A	
	SMG1	
	LOC100848906	
	CEP170B	
	UGT8	
	UPK2	
	XRCC5	
	ARHGDIG	
	TCRA	
	HYI	
	NAA30	
	KREMEN2	
	TUBGCP5	

	TP53I3	
	FAXC	
	PRKCZ	
	ALAS2	
	DOCK11	
	CSE1L	
	KCNQ3	
	DERA	
	HSD11B1	
	ASB6	
	BORCS8	
	DOP1A	
	HOXB3	
	NELFA	
	BBS7	
	ARHGAP32	
	C8H9orf40	
	ZCWPW1	
	LIX1	
	APH1B	
	NCK2	

	JOSD1	
	EIF2S1	
	HBA	
	ST3GAL3	
	HYAL1	
	ZC3H18	
	PYGM	
	MRPS2	
	OIT3	
	OCEL1	
	ADIPOQ	
	MTPAP	
	PFKFB2	
	ZBTB21	
	KRT6B	
	UBE2D3	
	ZNF77	
	POLR3GL	
	RECQL	
	RAB12	
	PTH1R	

	PRLH	
	RBP3	
	RASGRP1	
	TEFM	
	DHRS13	
	IGSF1	
	CENPT	
	DNAJC3	
	CD9	
	CD8B	
	EFHC2	
	TPD52L2	
	SEPTIN2	
	WBP11	
	ENGASE	
	SLC37A2	
	ESYT1	
	MRAS	
	LHCGR	
	DSP	
	LOC521383	

	MTHFD2L	
	APLN	
	GFI1B	
	LOC616942	
	CLDN4	
	FZD2	
	GPR137	
	UNK	
	MVP	
	METTL21E	
	GOLGA2	
	PNMT	
	CXCR5	
	IQSEC1	
	ZNF710	
	KPNA4	
	DEXI	
	RNF38	
	WNT5A	
	PTGER3	
	STAC3	

	CHKA	
	GOLGA3	
	PRR32	
	SLC52A3	
	ABR	
	CPNE1	
	SSPO	
	TENT2	
	MTMR4	
	CTDSPL	
	MARS1	
	HDAC1	
	PDLIM1	
	NOP10	
	YBX2	
	BRMS1L	
	FUBP1	
	KCTD21	
	MYL2	
	RPN2	
	DECR2	

	INSYN1	
	SQLE	
	LOC101902972	
	TNFRSF4	
	EPO	
	MAP9	
	LYPD8	
	MRPL16	
	SYNJ2BP	
	CCNY	
	CAPN8	
	CADM2	
	HYAL3	
