# Supplementary material for: Multi-Layer Transcriptome Analysis Uncovers Molecular Signatures of Coliform Mastitis in Lactating Cows
Source: Int J Mol Sci. 2026 Aug 9;27(16):7134. doi: 10.3390/ijms27167134 (PMC13513401; doi:10.3390/ijms27167134)
Supplement: Supplementary file 1 [file ijms-27-07134-s001.zip › Supplementary_Table S2.pdf]

Table S2: Network centrality measures (degree, closeness, betweenness) of hub genes identified in Blue, Turquoise, and Yellow modules.

Node_name	Degree	Closeness	Betweenness
**Blue Module**			
CLTC	18	91.3619	13118.78044
PIK3R1	14	85.4869	4129.86108
BRAF	12	81.56786	3227.166
SNW1	12	77.06905	3188.14422
HNRNPA2B1	11	80.82738	3693.27382
HSP90AA1	10	87.15952	6525.12034
PRPF40A	10	81.42381	3586.24338
SBNO1	10	77.25238	3621.91092
IGF2R	10	77.54167	5673.34027
LATS1	10	76.66667	3087.25064
KDM6B	9	77.08214	5284.42285
LARP4	9	75.33571	3282.99942
KRAS	16	88.7631	7791.85138
JUN	14	83.17262	5671.81145
**Turquoise Module**			
GAPDH	49	200.40281	102680.30762
STAT1	39	187.2294	35294.03097

STAT3	30	181.43535	15136.46781
CD44	26	176.525	20608.19668
TP53	24	179.62583	62174.13962
MMP9	22	167.73413	16107.70805
**Yellow Module**			
HSP90B1	11	41.702380952381	2313.20952380952
GMPPB	10	40.2412698412699	2063.92626262626
PSPH	8	41.0142857142858	1798.04415584416
HMGCL	7	39.602380952381	1729.07965367965
